# Multi-omics analysis the effects of Dhx37 deficiency on testis development and nucleolar homeostasis

**DOI:** 10.1038/s41420-025-02875-1

**Published:** 2026-01-14

**Authors:** Yuqing Jiang, Jiali Chen, Yanshuang Ren, Wenyuan Peng, Wanjun Shen, Yingyu Zhang, Jie Liu, Liujun Fu, Liping Li, Yujin Ma, Hongwei Jiang, Huifang Peng

**Affiliations:** 1 Henan Key Laboratory of Rare Diseases, Endocrinology and Metabolism Center, The First Affiliated Hospital, and College of Clinical Medicine of Henan University of Science and Technology, Luoyang, China; 2 National Clinical Research Center for Kidney Diseases, State Key Laboratory of Kidney Diseases, Beijing Key Laboratory of Kidney Disease Research, Nephrology Institute of the Chinese People’s Liberation Army, First Medical Center of Chinese PLA General Hospital, Beijing, China

**Keywords:** Sequencing, Endocrine reproductive disorders

## Abstract

The testicular microenvironment, with Sertoli cells as a key component, plays a pivotal role in spermatogenesis. *DHX37*, a member of the DEAH-box family of RNA helicases, has been identified as a pathogenic gene in 46, XY disorders of sex development (DSD), underscoring its potential significance in testicular development. Here, we focus on elucidating the role of *Dhx37* in maintaining Sertoli-cell survival. RIP-seq and RNAi-RNA-seq reveal that *Dhx37* safeguards nucleolar integrity and PI3K–AKT signaling, suppresses p53-driven apoptosis, and its loss triggers pro-apoptotic splicing. Cell-specific *Dhx37* knockout mice (*Dhx37*^−/−^) were subsequently generated to investigate the function of *Dhx37* in testicular development. In the *Dhx37*^−/−^ mice, we observed pronounced defects, including diminished testicular volume, lower testosterone levels, and marked vacuolization of the seminiferous tubules. Immunofluorescence staining revealed disruptions in both Sertoli and germ cell compartments, characterized by reduced cell proliferation and elevated apoptosis. The snRNA-seq disclosed marked changes in the expression of genes governing apoptosis and proliferation, findings that were further validated through qRT-PCR and Western blotting. In this study, we identified *Dhx37* as a pivotal determinant of nucleolar architecture in murine testicular Sertoli cells. Preservation of the nucleolus safeguards supporting normal testicular morphogenesis.

**Figure:**
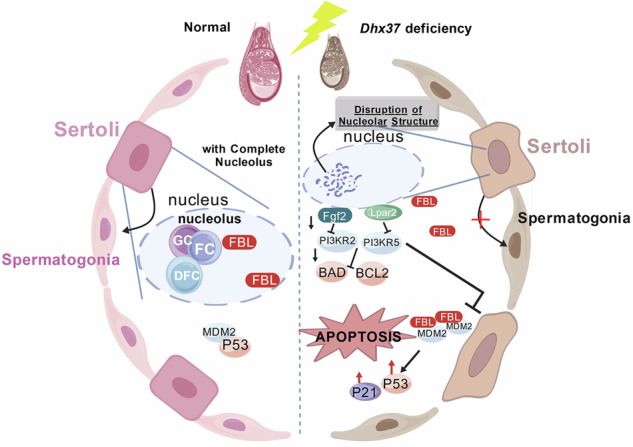
Graphical Abstract Schematic illustrating the proposed mechanisms by which *Dhx37* deficiency affects testicular development and spermatogenesis. In normal testes (left), Sertoli cells maintain a well-organized nucleolus with intact nucleolar structures, including Granular Component (GC), Fibrillar Center (FC), Dense Fibrillar Component (DFC). In this context, MDM2 interacts with P53, preventing the accumulation of P53 and inhibiting apoptosis, thereby supporting normal testicular architecture and spermatogenesis. However, in *Dhx37*^−/−^ mice (right), testicular volume is reduced, and seminiferous tubules undergo atrophy due to nucleolar stress in Sertoli cells. The disruption of nucleolar structure leads to the export of FBL from the nucleolus, where it binds to MDM2. This disruption is accompanied by downregulation of key factors in the PI3K pathway (Fgf2, Lpar2, PI3KR2, PI3KR5) and upregulation of the P53 pathway, culminating in apoptosis. As a result, *Dhx37* deficiency impairs Sertoli cell function, leading to a failure in supporting testicular development and spermatogenesis. Created with BioGDP.com.

Graphical Abstract Schematic illustrating the proposed mechanisms by which *Dhx37* deficiency affects testicular development and spermatogenesis. In normal testes (left), Sertoli cells maintain a well-organized nucleolus with intact nucleolar structures, including Granular Component (GC), Fibrillar Center (FC), Dense Fibrillar Component (DFC). In this context, MDM2 interacts with P53, preventing the accumulation of P53 and inhibiting apoptosis, thereby supporting normal testicular architecture and spermatogenesis. However, in *Dhx37*^−/−^ mice (right), testicular volume is reduced, and seminiferous tubules undergo atrophy due to nucleolar stress in Sertoli cells. The disruption of nucleolar structure leads to the export of FBL from the nucleolus, where it binds to MDM2. This disruption is accompanied by downregulation of key factors in the PI3K pathway (Fgf2, Lpar2, PI3KR2, PI3KR5) and upregulation of the P53 pathway, culminating in apoptosis. As a result, *Dhx37* deficiency impairs Sertoli cell function, leading to a failure in supporting testicular development and spermatogenesis. Created with BioGDP.com.

## Introduction

In mice, the transition of somatic gonadal cells within the mesoderm-derived urogenital ridge into Sertoli cells constitutes a pivotal event in testis differentiation [1]. Between embryonic day (E) 10 and E11, these cells remain bipotential, able to commit to either the ovarian or testicular lineage [2]. The onset of sex-determining region Y (SRY) expression at E11.5 redirects the gonadal somatic program toward a pre-Sertoli cell fate [3, 4]. These cells subsequently aggregate to form testicular cords. As the principal cellular constituent of the BTB, Sertoli cells facilitate spermatogenesis through intricate cellular interactions [5, 6]. They also secrete multiple growth factors, notably glial cell line-derived neurotrophic factor (GDNF) and fibroblast growth factor 2 (FGF-2) [7]. These ligands are pivotal for regulating the self-renewal of spermatogonial stem cells (SSCs) and the meiotic processes in spermatogonia [8]. Sertoli cells are the principal determinant of testicular volume. During the prepubertal period, Sertoli cells constitute the predominant testicular cellularity and actively proliferate [9]. The extent of this prepubertal proliferation fixes the adult Sertoli-cell population size and therefore limits ultimate testis volume [10]. Hence, Sertoli-cell proliferation is indispensable for establishing and maintaining normal testicular function [11].

The nucleolus, a central nuclear substructure, coordinates the transcription of rRNA and the assembly of ribosomes [12, 13]. Its structural and functional integrity is imperative for cellular proliferation and metabolic processes [14]. As an RNA helicase, *Dhx37* participates in cell growth and small ribosomal subunit pre-rRNA, both of which are essential for nucleolar homeostasis [15, 16]. Highly expressed in the male germ and Sertoli cells, *DHX37* has been identified as the causative gene for 46, XY DSD [17, 18]. Although *Dhx37* expression is evident in the testes, the precise molecular pathways by which it governs testicular development and drives gonadal dysgenesis remain elusive [19–21].

In this study, we generated Sertoli cell-specific *Dhx37* conditional knockout mice by crossing *Dhx37*^flox/flox^ animals with Amh-Cre transgenic mice, in which Cre recombinase is driven by the Amh promoter. Morphometric analysis demonstrated that the testes of *Dhx37*^−/−^ mice were significantly reduced in size and weight compared to those of wild-type (WT) counterparts. Immunofluorescence and electron microscopy were employed to examine alterations in the testicular somatic and germ cells populations and to assess nucleolar structure in Sertoli cells. Collectively, these data underscore a pivotal role for *Dhx37* in preserving Sertoli cell nucleolar integrity, supporting Sertoli cell viability, and thereby sustaining a permissive microenvironment for spermatogenesis.

## Result

### Integrative re-analysis of public single-cell data and RIP-seq uncovers *Dhx37* expression dynamics and regulatory pathways

**Fig. 1:**
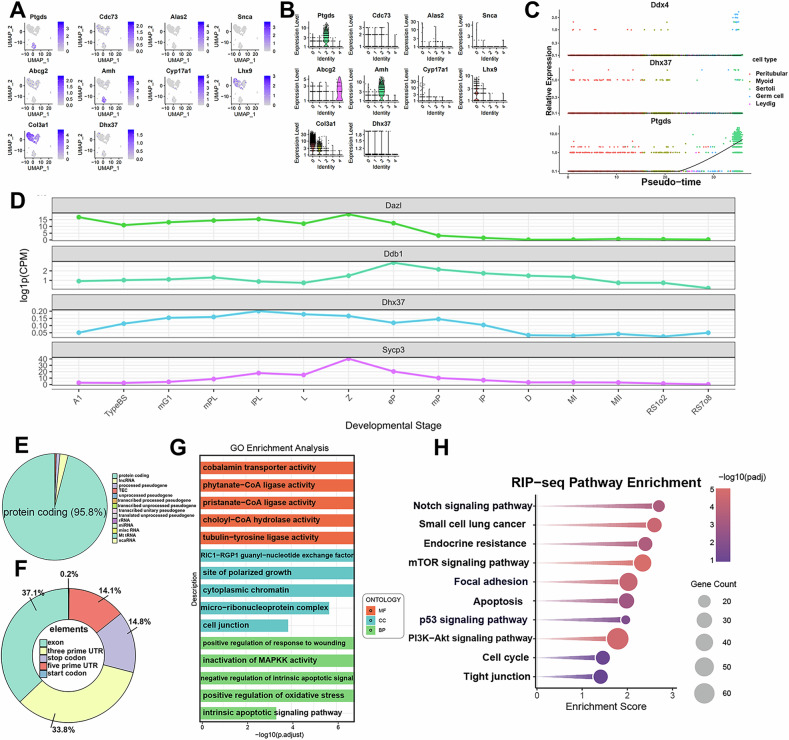
*Dhx37* is broadly expressed during murine spermatogenesis and preferentially binds mRNAs that regulate apoptosis, cytoskeletal dynamics and cell-junction integrity. **A** The expression distribution of different genes (*Ptgds*, *Cdc73*, *Alas2*, *Snca*, *Abcg2*, *Amh*, *Cyp17a1*, *Lhx9*, *Col3a1*, and *Dhx37*) in the UMAP dimensionality reduction space for 3 postnatal day mice. Each subplot represents the expression level of a specific gene, with colors ranging from gray to blue indicating low to high gene expression levels. UMAP_1 and UMAP_2 are the two dimensions after UMAP reduction. Data from the GEO database (GSE130554). **B** Single cell gene expression levels of *Ptgds*, *Cdc73*, *Alas2*, *Snca*, *Abcg2*, *Amh*, *Cyp17a1*, *Lhx9*, *Col3a1*, and *Dhx37* across various cell clusters in testis from P3.5. Each panel represents a specific gene with expression levels plotted on the y-axis and cell identities on the x-axis. **C** The relative expression levels of three genes (*Ddx4*, *Dhx37*, and *Ptgds*) in different cell types within mouse testicular tissue. The data are presented as scatter plots, with the horizontal axis representing pseudotime and the vertical axis representing relative expression levels. **D** Line plots of log10 counts-per-million (CPM) for *Dazl*, *Ddb1*, *Dhx37* and *Sycp3* across spermatogenic stages: A1 (type A1 spermatogonia), typeBS (type B spermatogonia), mG1 (G1 phase of early spermatocytes), mPL (mid-S phase of preleptotene spermatocytes), IPL (late S phase of preleptotene spermatocytes), L (leptotene stage in meiotic prophase I), Z (zygotene stage), eP (early pachytene stage), mP(mid-pachytene stage), IP (intermediate pachytene stage), D (diplotene stage), MI (metaphase I of meiosis I), MII (metaphase II of meiosis II), RS1o2 (spermatids at steps 1–2), RS7o8 (spermatids at steps 7–8). Data reanalyzed from Chen et al. [22]. **E** Pie chart showing transcript biotypes harboring *Dhx37* RIP-seq peaks in TM4 cells; 95.8% map to protein-coding mRNAs. **F** Distribution of peaks across mRNA features: exons (37.1%), 3′ UTRs (33.8%), 5′ UTRs (14.8%), stop/start codons (14.1%) and introns (0.2%). **G** GO enrichment of *Dhx37*-associated transcripts (Benjamini–Hochberg-adjusted q < 0.05); bars represent −log10 q and are colored by ontology category (BP, CC, MF). **H** KEGG pathway enrichment dot-plot; dot size indicates number of *Dhx37*-bound genes and color encodes −log10 Padj. Top terms include focal adhesion, apoptosis, p53 signaling and PI3K–Akt signaling, linking *Dhx37* to stress-response and pro-survival pathways.

We reanalyzed publicly available single-cell RNA-seq data from GEO and applied dimensional reduction and trajectory inference approaches to quantify *Dhx37* transcripts in murine testes at P3.5. Our analysis revealed ubiquitous *Dhx37* expression across all cell clusters, albeit at lower transcript abundance than canonical markers including *Amh*, *Star*, and *Mvh* (Fig. 1A, B). Notably, pseudotime modeling did not implicate *Dhx37* as a driver of lineage progression (Fig. 1C).

Using the publicly available single-cell RNA sequencing (scRNA-seq) dataset of mouse spermatogenesis generated by Chen et al., we systematically profiled the expression dynamics of *Dazl*, *Ddb1*, *Dhx37*, and *Sycp3* across discrete developmental stages (Fig. 1D) [22]. The germ-cell marker *Dazl* remained relatively stable, exhibiting a modest peak in type B spermatogonia, which is consistent with its established role in early germ-cell differentiation. By contrast, the DNA repair gene Ddb1 was markedly down-regulated in late round spermatids, indicating a reduced requirement for DNA repair during terminal differentiation. Notwithstanding the overall decline, the similarity between *Dhx37* and *Dazl* trajectories suggests that *Dhx37* participates in early-stage cellular programs and fulfills a broad developmental role that is not restricted to meiosis.

RIP-seq peaks map overwhelmingly to protein-coding RNAs (95.8%), with only minor representation of rRNA, lncRNA, and pseudogenes (Fig. 1E). Binding sites concentrate in exons (37.1%), and 3′ UTRs (33.8%), followed by 5′ UTRs (14.8%), stop/start codons (14.1%), and introns (0.2%), indicating a preference for mature mRNAs and their regulatory termini (Fig. 1F).

RIP-seq in TM4 Sertoli cells showed that *Dhx37*-bound transcripts are significantly enriched in pathways governing pivotal cellular processes. GO analysis terms cluster around intrinsic apoptotic signaling and oxidative stress response, suggesting *Dhx37* contacts transcripts involved in stress adaptation and death programs (Fig. 1G). KEGG pathway analysis of *Dhx37*-associated transcripts revealed significant enrichment for the focal adhesion, apoptosis, and p53 signaling pathways, implicating the helicase in cytoskeletal regulation and cell death (Fig. 1H). A concomitant enrichment of the PI3K–Akt cascade links *Dhx37* to stress-adaptive, pro-survival signaling, while the over-representation of the tight-junction pathway suggests a role in maintaining Sertoli–and Sertoli–germ cell contacts.

### RNAi-based *Dhx37* knockdown: RNA-seq and alternative splicing analysis

**Fig. 2:**
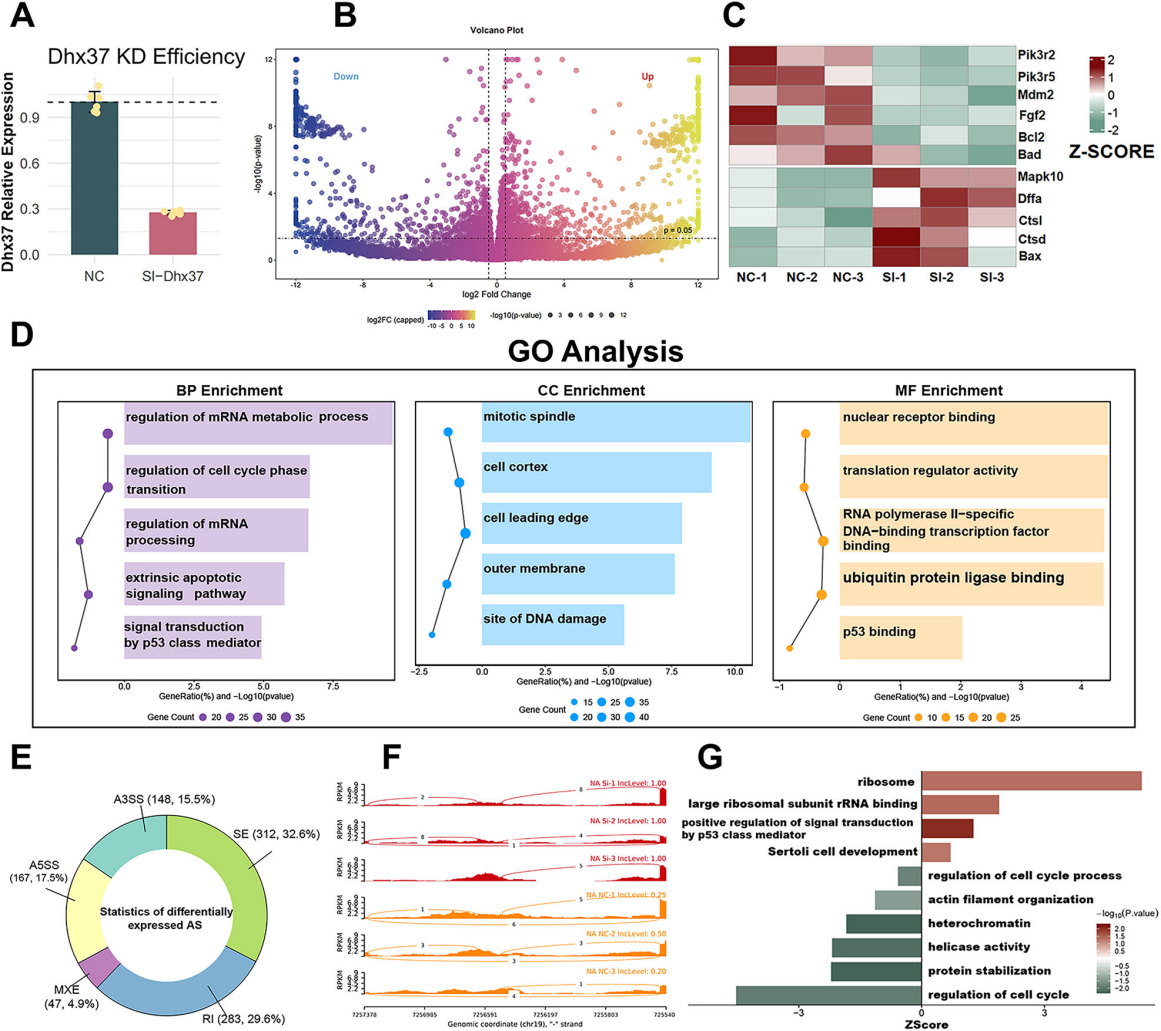
Transcriptomic and post-transcriptional consequences of *Dhx37* silencing in TM4 cells. **A** qRT-PCR confirmation of *Dhx37* knockdown efficiency. Relative mRNA abundance is normalized to GAPDH and presented as mean ± SD (n = 3 biological replicates per group). ***P < 0.001, two-tailed Student’s *t* test versus NC. **B** Volcano plot of DEGs identified by bulk RNA-seq (n = 3 per group). Genes with |log₂ fold-change| > 1 and Benjamini–Hochberg adjusted P < 0.05 are colored yellow (up-regulated) or blue (down-regulated). **C** Heat-map (row Z-scores) of canonical PI3K–Akt and apoptosis-related transcripts across individual NC and *Dhx37*-SIRNA replicates. **D** GO over-representation analysis of up-regulated DEGs. The five most significant terms in each category—biological process (BP, purple), cellular component (CC, blue) and molecular function (MF, orange)—are shown. Dot size denotes gene count, and dot position reflects −log_10_ (q-value). **E** Summary of 949 significant alternative-splicing (AS) events detected by rMATS (FDR < 0.05, |ΔPSI| ≥ 0.1). Sector size is proportional to event number. A3SS, alternative 3′ splice site; A5SS, alternative 5′ splice site; SE, skipped exon; RI, retained intron; MXE, mutually exclusive exon. **F** Representative sashimi plots highlighting *Dhx37*-dependent exon-skipping, intron-retention and splice-site shifts in selected loci (mm10 coordinates; read coverage scale identical within each track). **G** GO enrichment of genes harboring significant AS events. Bars are ordered by Z-score; color scale indicates −log₁₀ P-value. Terms linked to ribosome biogenesis, p53 signaling and cell-cycle regulation are prominently enriched.

To investigate the cell-autonomous roles of *DHX37* in testicular somatic (Sertoli) cells, we generated an in vitro *Dhx37* knockdown model in TM4 murine Sertoli cells and performed bulk RNA-seq profiling (Fig. 2A, B). GO enrichment of up-regulated differentially expressed genes (DEGs) underscored programs tightly associated with apoptosis and stress signaling, including “signal transduction by p53 class mediator,” “extrinsic apoptotic signaling pathway,” and “regulation of cell-cycle phase transition.” (Fig. 2D). Notably, the category “positive regulation of signal transduction by p53 class mediator” was strongly enriched. KEGG analysis of down-regulated DEGs revealed PI3K–Akt pathways (Supplementary Fig. 1A). Transcriptomic signatures indicated a coherent pro-apoptotic shift, with simultaneous activation of p53 signaling and attenuation of the pro-survival PI3K–Akt axis. Heat-map inspection demonstrated down-regulation of core PI3K–Akt components (Pik3r2, Pik3r5), upstream growth cues (Fgf2), and anti-apoptotic effectors (Mdm2, Bcl2) (Fig. 2C). Diminished Mdm2 expression provides a mechanistic basis for p53 stabilization. Conversely, apoptotic effectors were markedly induced, including death-receptor signaling (Fas), stress-activated MAPK (Mapk10/JNK3), DNA-fragmentation machinery (Dffa), and the canonical p53 target Bax.

To assess post-transcriptional consequences, we profiled alternative splicing (AS) events. *Dhx37* KD triggered 949 significant AS events (Fig. 2E). Sashimi plots corroborated *Dhx37*-dependent alterations in exon usage and intron retention (Fig. 2F). AS-affected genes were enriched in cell-cycle regulation, DNA damage response, and p53-linked apoptotic signaling, reinforcing DEG-derived conclusions (Fig. 2G).

In summary, our multi-omics analyses suggest that *Dhx37* deficiency may compromise nucleolar integrity and ribosome biogenesis, possibly activate the RPL11–MDM2–p53 axis, attenuate PI3K–Akt survival signaling, and shift splicing program interrelated perturbations that could together promote a pro-apoptotic state in cells.

### Spatiotemporal expression of *Dhx37* in mice testis and generation of Sertoli-cell-specific *Dhx37* knockout

**Fig. 3:**
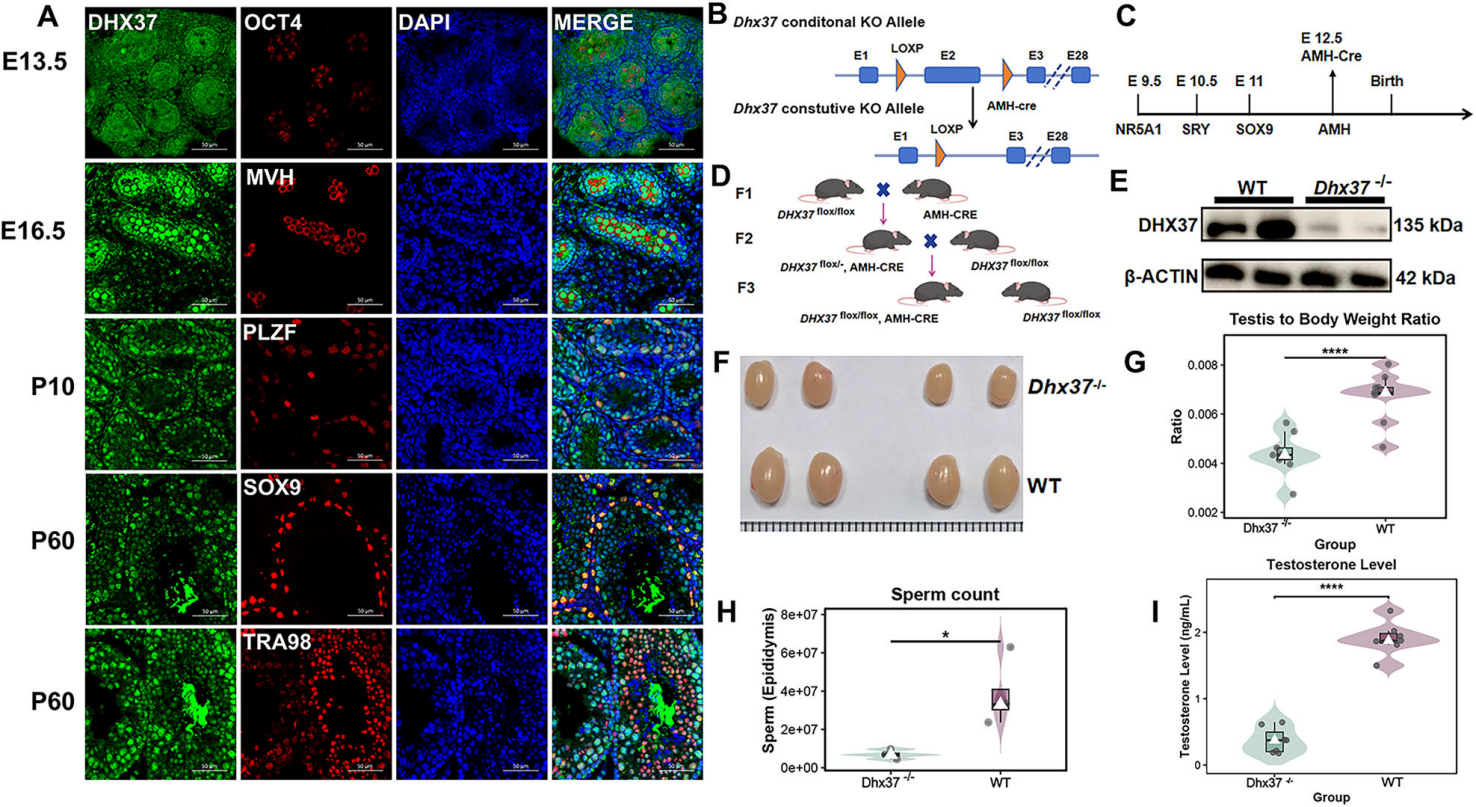
Localization and functional role of *DHX37* during testicular development and construction of conditional knockout mice. **A** Immunofluorescence localization of DHX37 (green) during testis development. Co-staining was performed with stage-restricted markers (red): OCT4 (primordial germ cells, E13.5), MVH (germ-cell lineage, E16.5), PLZF (spermatogonial stem cells, P10), SOX9 (Sertoli-cells, P60) and TRA98 (all germ-line cells, P60). Nuclei were counter-stained with DAPI (blue). Scale bars, 50 μm. **B** Targeting strategy. The floxed *Dhx37* allele carries loxp sites flanking exon 2. **C** Developmental timing of Cre activity. AMH-Cre expression initiates at embryonic day 12.5, after male-sex determination but before Sertoli-cell maturation. **D** Breeding scheme used to obtain Sertoli-cell-specific *Dhx37* knock-out mice (*Dhx37*^*flox*/flox^, AMH-Cre, hereafter *Dhx37*^−/−^) and WT littermates. **E** Gross morphology of testes from 8-week-old *Dhx37*^−/^^−^ and WT males. Scale bar, 5 mm. **F** WB of cells confirming near-complete loss of DHX37 protein in *Dhx37*^−/−^ animals. β-ACTIN served as loading control. **G** Testis-to-body-weight ratio is markedly reduced in *Dhx37*^−/−^ males (n = 9) compared with WT (n = 9). Violin plots show median ± inter-quartile range. ****P < 0.0001, two-tailed unpaired Student’s *t* test. **H** Epididymal sperm counts are significantly lower in *Dhx37*^−/−^ males (n = 4) than in WT controls (n = 4). *P < 0.05, two-tailed unpaired Student’s *t* test. **I** Serum testosterone concentrations measured by ELISA (n = 7 mice per group). ****, P < 0.0001.

By E13.5 and E16.5, when testicular differentiation is largely complete, DHX37 exhibited broad expression throughout the developing testis, rather than being confined to OCT4/MVH-positive primordial germ cells. And at P10, DHX37 expression was robust in PLZF-positive spermatogonial stem cells, suggesting its key role in the proliferative expansion phase of testicular development. By P60, DHX37 was detected in both SOX9-positive Sertoli cells and TRA98-positive post-meiotic germ cells, with a predominantly nuclear distribution, highlighting its crucial role in both germ cells and Sertoli cells, contributing significantly to testicular development across these stages (Fig. 3A). Their significance in both the early development and ongoing function of the testis emphasizes the importance of *Dhx37* in Sertoli cells. These findings demonstrate that *Dhx37* is consistently expressed throughout testis development.

Due to the essential role of Sertoli cells in both testicular development and spermatogenesis, we generated a Sertoli-cell-specific *Dhx37* knockout by crossing *Dhx37* ^flox/flox^ mice with AMH-Cre mice (Fig. 3A). The AMH-Cre transgene activates Cre recombinase at E12.5, prior to Sertoli-cell maturation (Fig. 3B, C). The F3 progeny carrying *Dhx37* ^flox/flox^; AMH-Cre (referred to as *Dhx37* ^−/−^) were compared to WT (Fig. 3D). Western blotting confirmed efficient recombination, showing an almost complete loss of DHX37 protein in Sertoli cells (Fig. 3E). Morphological assessment revealed significantly smaller testes in males compared with WT (Fig. 3F). Quantitative analysis demonstrated a pronounced reduction in the testis-to-body-weight ratio in *Dhx37* ^−/−^ mice (Fig. 3G; ****P < 0.0001). Consistent with impaired testis volume, epididymal sperm counts were also markedly lower in mutants (Fig. 3H; *P < 0.05). *Dhx37*^−/−^male mice exhibited significantly reduced sperm counts and were infertile, as evidenced by their failure to produce offspring when mated with wild-type female mice. These findings indicate that Sertoli cell-specific knockout of *Dhx37* leads to male infertility.

### Impaired testicular architecture and steroidogenesis revealed by histological and immunohistochemical analyses

**Fig. 4:**
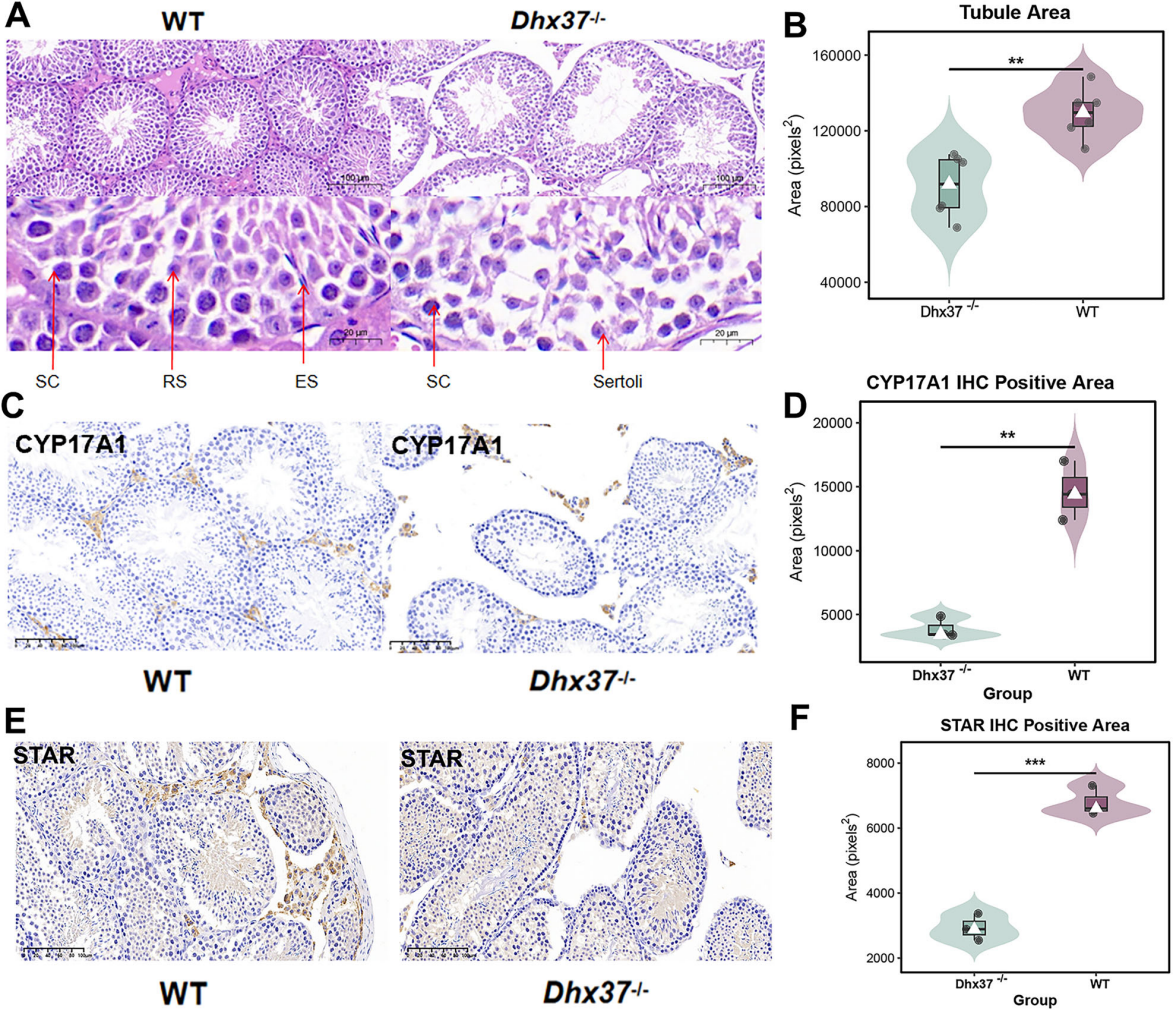
*Dhx37* deficiency compromises seminiferous-tubule integrity and Leydig-cell steroidogenesis. **A** Representative H&E staining of adult testes from WT and *Dhx37*^−/−^ mice. *Dhx37*^−/−^ tubules display marked atrophy, luminal vacuolization and loss of elongating spermatids (ES); round spermatids (RS) and spermatocytes (SC) are indicated by red arrows. Scale bars, 50 µm (upper panels) and 20 µm (lower panels). **B** Violin plot of total seminiferous-tubule cross-sectional area quantified in ImageJ (four mice per genotype, 50 tubules per mouse). **, P < 0.01 (unpaired two-tailed t-test). **C** IHC for the steroidogenic enzyme CYP17A1 in WT and *Dhx37*^−/−^ testes. Brown staining marks Leydig cells, scale bar = 100 µm. **D** Quantification of CYP17A1-positive area (n = 3 mice per genotype, ≥10 fields per mouse). **, P < 0.01. **E** IHC for the cholesterol-transport protein STAR in adjacent sections; scale bar = 100 µm. **F** Quantification of STAR-positive area (n = 3 mice per genotype). ***, P < 0.001.

H&E staining demonstrated marked atrophy and vacuolization of seminiferous tubules in *Dhx37*^−/−^ testes, which was accompanied by significant reductions in serum testosterone concentrations (Figs. 3I and 4A). Quantitative ImageJ analysis of H&E sections revealed a pronounced decrease in the total seminiferous-tubule area in *Dhx37*^−/−^ testes, indicative of compromised testicular architecture (Fig. 4B). To elucidate the basis of the lower testosterone output, we performed immunohistochemistry for the steroidogenic enzymes CYP17A1 and STAR, both of which are indispensable for androgen biosynthesis (Fig. 4C, E). *Dhx37*^−/−^ testes exhibited a significant decrease of CYP17A1 and STAR immunoreactivity relative to WT (Fig. 4D, F). Collectively, these data indicate that loss of *Dhx37* disrupts Leydig cell steroidogenesis, thereby contributing to the testosterone deficit observed in *Dhx37*^−/−^ males.

### *Dhx37* deletion results in the decrease of Sertoli and germ cells in adult male mice

**Fig. 5:**
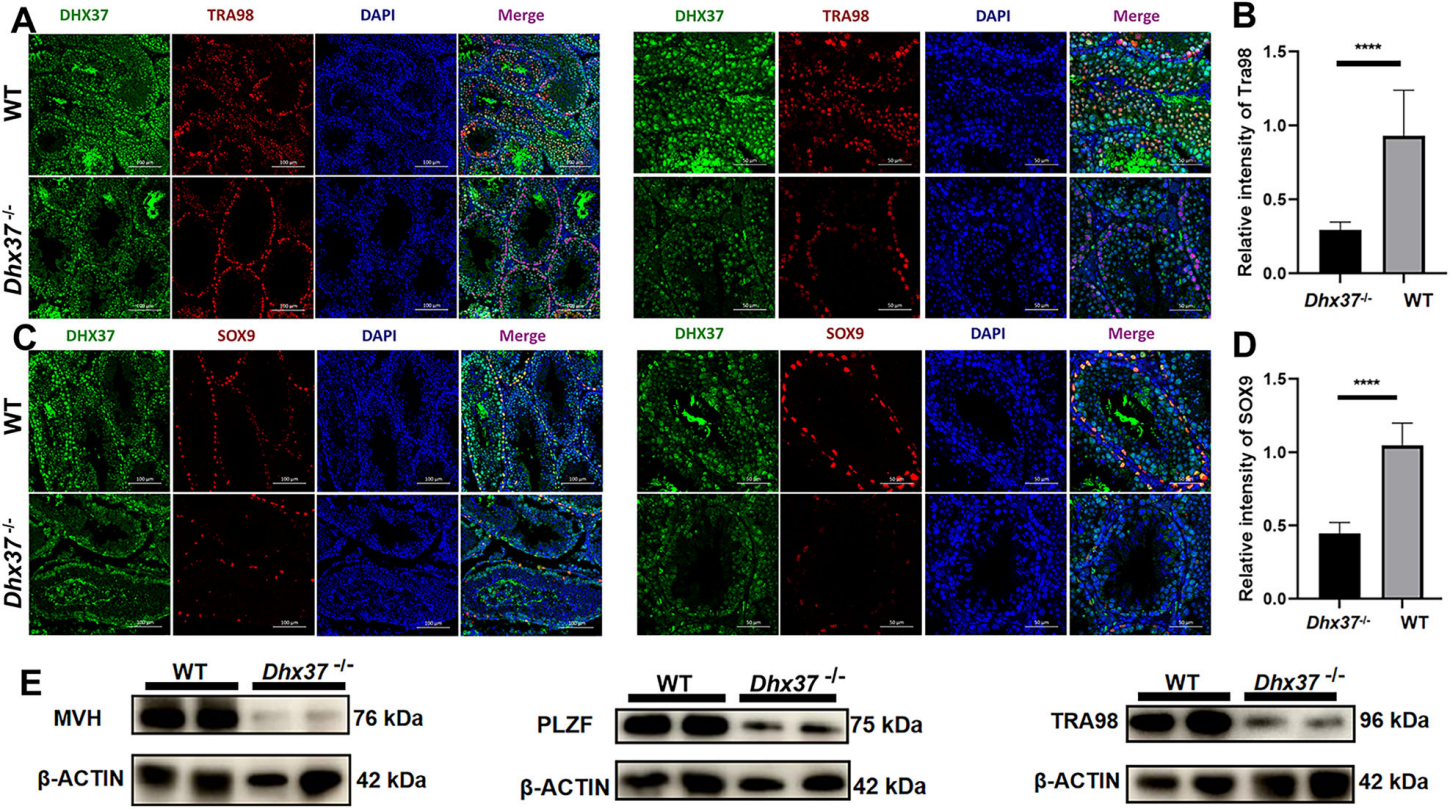
Loss of *Dhx37* depletes germ- and Sertoli-cell populations in adult mouse testes. **A** Double immunofluorescence for DHX37 (green) and the pan-germ-cell marker TRA98 (red) in WT and *Dhx37*^−/−^ sections; nuclei, DAPI (blue). For each genotype, representative low (100 μm) and high (50 μm) magnification panels show matched fields. **B** Quantification of TRA98 fluorescence intensity (n = 3). ****P < 0.0001 (unpaired two-tailed t-test). **C** Double staining for DHX37 (green) and the Sertoli-cell marker SOX9 (red) in adjacent sections. **D** Quantification of SOX9 signal (n = 3). ****P < 0.0001. **E** WB validation of germ-cell markers (MVH, PLZF, TRA98) and Sertoli-cell markers in whole-testis lysates. β-ACTIN serves as a loading control.

Then we conducted immunofluorescence staining followed by quantitative intensity analysis. Consistent with histological data, *Dhx37*^−/−^ testes displayed a significant decrease in TRA98-positive germ cells (Fig. 5A, B) and in SOX9-positive Sertoli cells (Fig. 5C, D) relative to wild-type littermates, resembling the gonadal dysgenesis reported in humans carrying *DHX37* variants. Western blot analysis corroborated these observations, revealing pronounced down-regulation of germ-cell markers MVH/VASA, PLZF, and TRA98 in *Dhx37*^−/−^ testes (Fig. 5E).

In summary, these data demonstrate that Sertoli-cell-specific loss of *Dhx37* precipitates profound reproductive deficits, including diminished testicular size, reduced sperm output and testosterone production, depletion of germ and Sertoli cell populations, and heightened apoptosis.

### snRNA-seq analysis reveals the effects of *Dhx37* knockout on gene expression in Sertoli cells

**Fig. 6:**
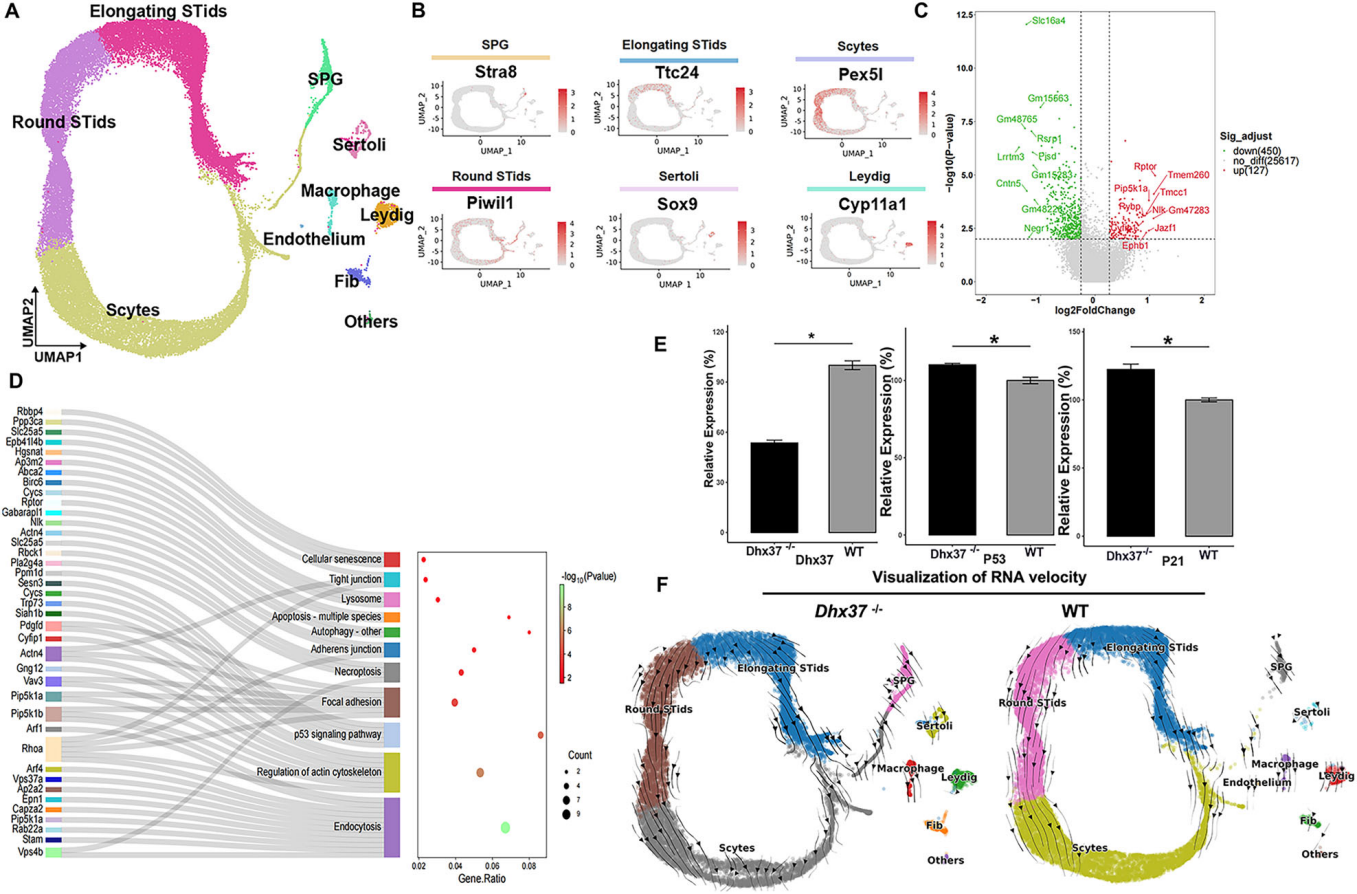
*Dhx37* loss remodels the Sertoli-cell transcriptome and perturbs germ-cell developmental flow. **A** UMAP embedding of single-nucleus RNA-seq profiles from P60 WT and *Dhx37*^−/^^−^ testes, colored by the ten cell identities resolved: spermatogonia (SPG), spermatocytes (Scytes), round spermatids (round STids), elongating spermatids (elongating STids), Leydig cells, cells, macrophages, endothelial cells, fibroblasts and others. **B** Feature plots illustrating representative markers for each cluster (e.g., Stra8 for SPG, Ttc24 for elongating spermatids). **C** Volcano plot of DEGs in *Dhx37*^−/^^−^ versus WT cells (two-sided Wald test, Benjamini–Hochberg false-discovery rate < 0.05; gray = nonsignificant, red = up-regulated, green = down-regulated). **D** Sankey diagram and dot-plot summarizing KEGG enrichment. Significantly over-represented pathways converge on membrane dynamics, actin-cytoskeleton remodeling and p53-mediated apoptotic signaling (dot size = gene ratio, color = –log 10 adjusted P). **E** Validation of selected stress/apoptosis markers: bar graphs show mean ± SD normalized mRNA counts for Dhx37, p53, and p21 in whole testis RNA sample (n = 3 biological replicates per genotype); P < 0.05, unpaired two-tailed t-test. **F** RNA-velocity vectors projected onto the UMAP for *Dhx37*^−/^^−^ and WT datasets. Loss of *Dhx37* increases directional dispersion within round-to-elongating spermatid transitions and in Sertoli-cell clusters, indicating destabilized transcriptional trajectories.

To investigate the regulatory mechanism of *Dhx37* in Sertoli cells, snRNA-seq analysis was performed on testicular cells isolated from whole testes of wild-type and *Dhx37*^−/−^ mice at P60 (Fig. 6A). After quality control, we obtained single-cell transcriptomes for both wild-type and *Dhx37*^−/−^ mice. Dimension reduction and visualization using uniform manifold approximation and projection (UMAP) allowed us to categorize the cells into ten distinct clusters: round spermatids, elongating spermatids, spermatocytes, spermatogonia, Leydig cells, fibroblasts, sertoli cells, macrophages, endothelial cells, and others, based on known marker genes (Fig. 6B). To further elucidate the effect of *Dhx37* loss on cellular responses in the testes, we focused on Sertoli cells for detailed analysis. Differentially expressed gene analysis identified 577 genes in the Sertoli cells of *Dhx37*^−/−^ mice, including 127 upregulated and 450 downregulated genes (Fig. 6C), indicating significant changes in gene expression profiles. Collectively, these pathways converge on the activation of membrane dynamics, cytoskeletal re-organization, and stress-induced apoptotic responses (Fig. 6D). Consistent with the pronounced enrichment of the p53 signaling pathway, the key effectors p53 and p21 are significantly up-regulated in *Dhx37*^−/−^ whole testis (P < 0.05; Fig. 6E), further supporting that *Dhx37* deletion triggers a p53-mediated apoptosis/senescence program.

We also investigated whether *Dhx37* deletion affects the differentiation trajectory of spermatogenic cells. Using scVelo, a model that assesses RNA splicing speed to predict the future transcriptional state of individual cells, we inferred the developmental trajectory of spermatogenesis based on snRNA-seq data. Notably, the *Dhx37* defect altered the direction of the velocity vector within spermatids. In the *Dhx37*^−/−^ group, RNA velocity between round spermatids and elongating spermatids showed greater variability, suggesting increased fluctuations in gene expression (Fig. 6F). Similarly, the RNA velocity of Sertoli cells in the *Dhx37*^−/−^ group exhibited greater variability and instability, indicating that *Dhx37* deletion disrupts normal gene expression and function in these cells.

**Fig. 7:**
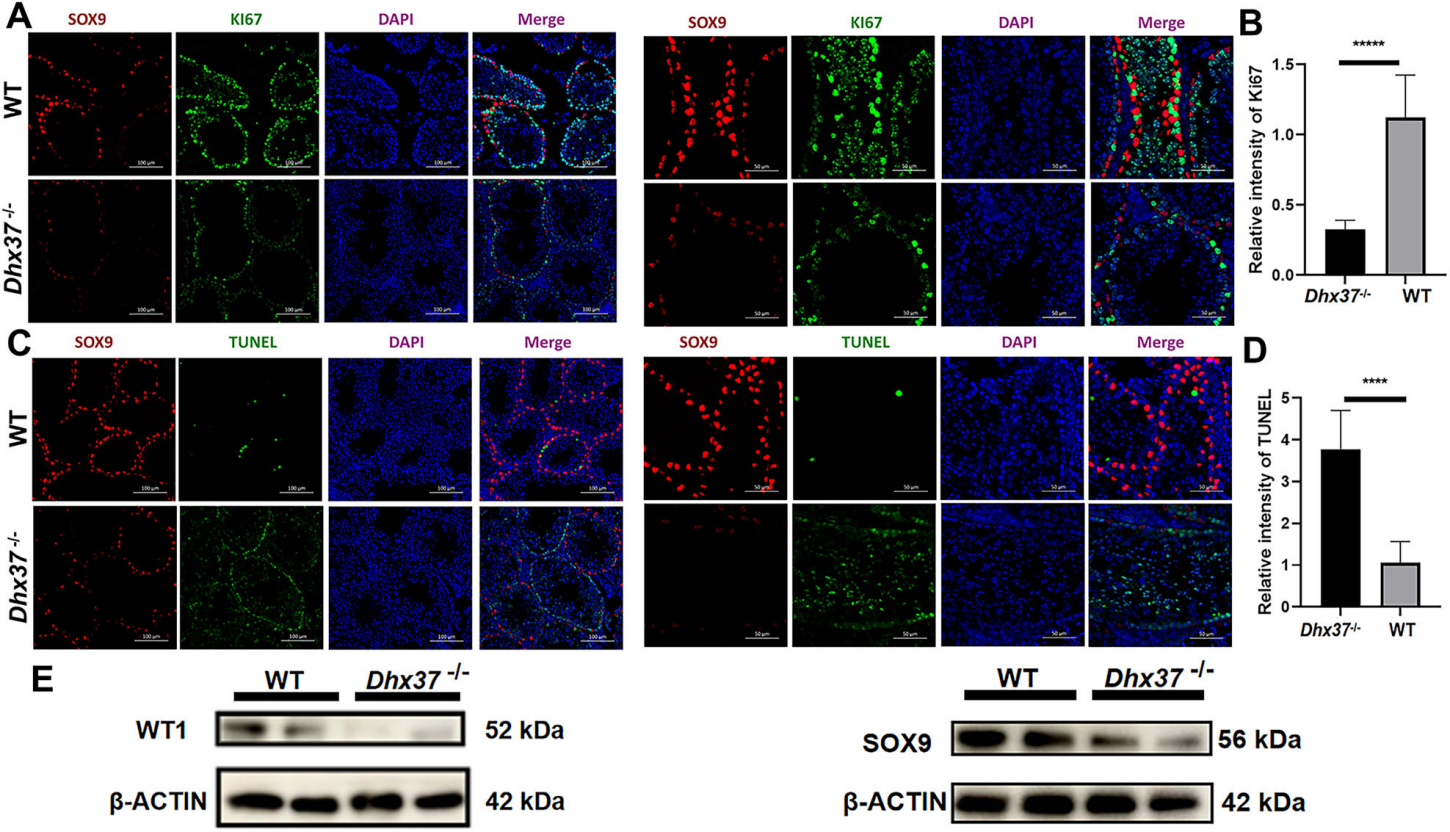
DHX37 loss in cells diminishes proliferative activity and enhances apoptosis in adult testes. **A** Immunofluorescence of adult (P60) testis sections stained for SOX9 (red, Sertoli-cell nuclei), KI67 (green, proliferating cells) and DAPI (blue). *Dhx37*^−/−^ tubules show a marked reduction of KI67-positive cells compared with WT controls. Scale bars, 100 μm (left sub-panel) and 50 μm. **B** Quantification of KI67 fluorescence intensity per tubule (mean ± SD; n = 3 mice per genotype). ****P < 0.0001, two-tailed unpaired Student’s *t* test. **C** TUNEL assay (green) combined with SOX9 immunostaining (red) demonstrates a pronounced increase in apoptotic cells in *Dhx37*^−/−^ testes. Scale bars as in (**A**). **D** Quantification of TUNEL signal (mean ± SD; n = 3 mice per genotype). ****P < 0.0001, two-tailed unpaired Student’s *t* test. **E** WB analysis of whole-testis lysates from adult WT and *Dhx37*^−/−^ males. Levels of the Sertoli-cell markers WT1 and SOX9 are reduced in *Dhx37*^−/−^ testes. β-ACTIN served as a loading control.

Meanwhile, by immunofluorescence staining with anti-ki67 antibody, we observed a marked attenuation of Sertoli proliferation in *Dhx37* mice (Fig. 7A, B). We also detected possible Sertoli apoptosis by TUNEL staining and found a significant increase in apoptotic cells in *Dhx37*^−/−^ testis tissues (Fig. 7C, D). The western blot confirmed the decrease of the Sertoli-cell markers WT1 and SOX9 within *Dhx37*^−/−^ testes (Fig. 7E). Taken together, these data suggest that *Dhx37* knockout disrupts the apoptosis signaling pathway and may further lead to phenotypic defects in Sertoli cell proliferation and survival.

### *Dhx37* deletion results in the depletion disorganized distribution of adhesion factors

**Fig. 8:**
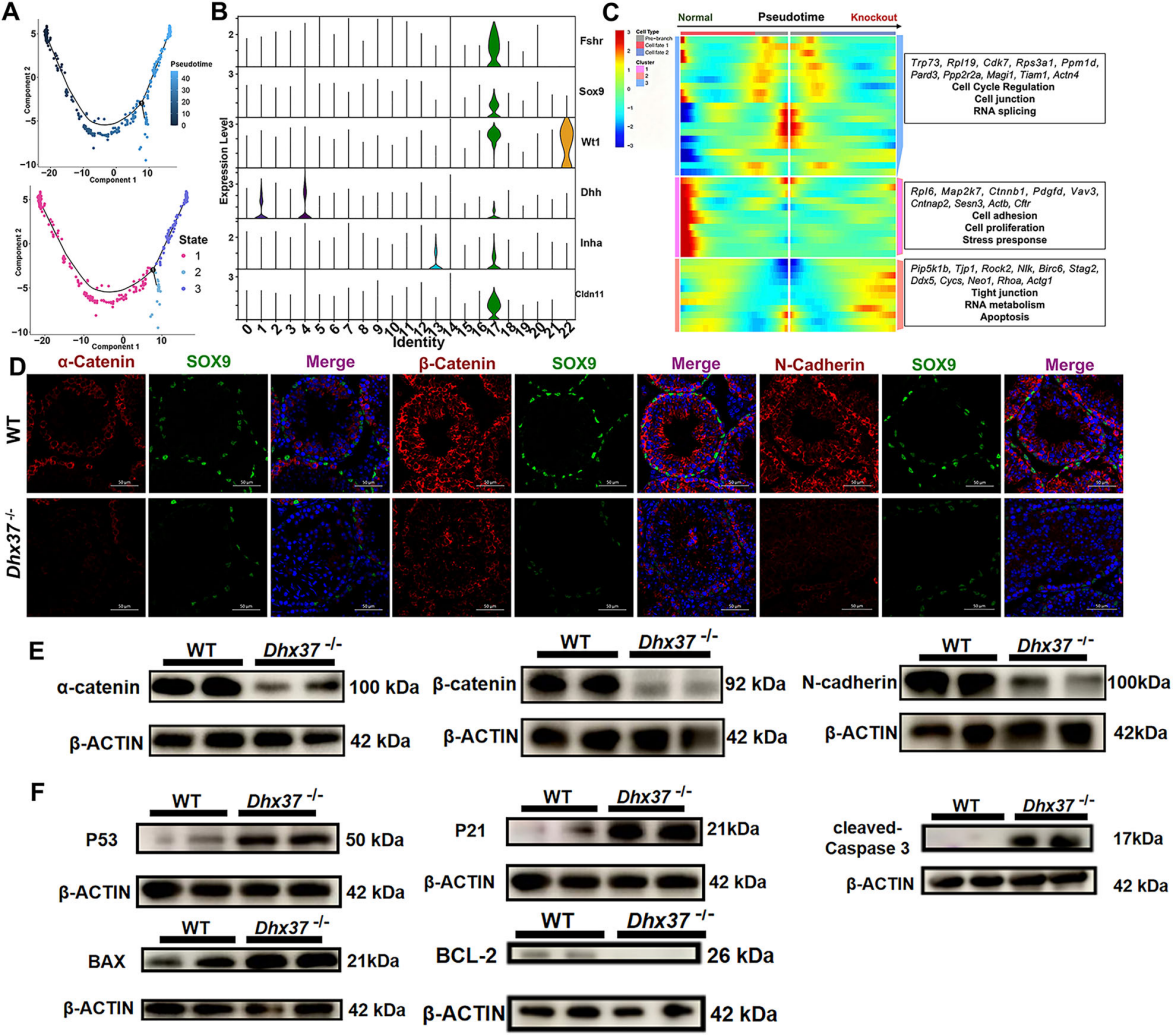
*Dhx37* deletion results disorganized distribution of adhesion factors and apoptosis. **A** Trajectory analysis of Sertoli cells using Monocle 2 revealed a pseudotime trajectory with one branching point, indicating distinct developmental states. **B** Expression dynamics of selected lineage- and adhesion-associated genes along the Sertoli cell trajectory. **C** Heatmap showing gene expression programs across pseudotime in wild-type (normal) and *Dhx37* knockout Sertoli cells, highlighting enrichment of cell cycle, adhesion, junctional remodeling, stress response, and apoptosis pathways. **D** Immunofluorescence staining of α-catenin, β-catenin, and N-cadherin (red) in combination with the Sertoli cell marker SOX9 (green) demonstrated disrupted adhesion molecule localization and reduced Sertoli cell numbers in *Dhx37*^−/−^ testes compared to WT. **E** Western blot analysis confirmed significant down-regulation of α-catenin, β-catenin, and N-cadherin in *Dhx37*^−/−^ testes relative to WT. **F** Western blot detection of apoptotic signaling components showed up-regulation of p53 and p21, and increased levels of pro-apoptotic markers BAX and cleaved caspase-3, alongside reduced BCL-2 in *Dhx37*^−/−^ Sertoli cell.

To define the identity and developmental hierarchy of Sertoli cells and their differentiating progenies, we applied trajectory analysis using Monocle 2. The results revealed the developmental order of the pseudotime trajectories with one branching point (Fig. 8A). We then examined changes in gene expression along the Sertoli cell trajectory to identify cell type signatures and candidate genes related to cell fate transitions (Fig. 8B). By analyzing cell-type-specific gene expression patterns, we identified genes and pathways enriched in Sertoli cells at early, middle, and late stages of development (Fig. 8C). The mature Sertoli-cell cluster showed strong expression of lineage-and barrier-associated transcripts, including *Trp73* and *Actn4*, with abundant ribosomal and cell-cycle genes (*Rpl19*, *Rps3a1*, *Cdk7*, *Ppm1d*). In addition, several regulators of tight-junction dynamics and apoptosis were upregulated, *notably Pip5k1b*, *Tjp1*, *ROCK2*, *RhoA*, *Actg1*, *Cycs*, and the adhesion receptor *Neo1*, implying intensified junctional remodeling and stress signaling under *Dhx37* deficiency. By contrast, cells occupying the early pseudotime branch were enriched for genes related to proliferation and adhesion, such as Rpl6, Map2k7, Ctnnb1, Pdgfd, Vav3, Cntnap2, Sesn3, Actb, and Cftr. Because both the abundance and functional competence of Sertoli cells govern the stability of adhesion complexes within the seminiferous epithelium, these transcriptional shifts are likely to compromise junction integrity and, consequently, spermatogenesis.

*Dhx37*^−/−^ mice exhibit reduced testicular mass, infertility, and altered Sertoli cell function. We examined three key cell adhesion factors (α-catenin, β-catenin, and N-cadherin) and found that their distribution was disrupted, adversely affecting the spermatogenic microenvironment. In WT mice, Sertoli cells, identified by SOX9 immunostaining, were extensively distributed along the periphery of the seminiferous tubules (Fig. 8D). In contrast, *Dhx37*^−/−^ testes showed a significant decrease in Sertoli cell populations within the seminiferous tubules (Fig. 7A–D). These findings collectively suggest that *Dhx37* knockout disrupts not only the proliferation of Sertoli cells but also alters the typical distribution of cell adhesion molecules associated with Sertoli cells, impairing the structural integrity of the seminiferous tubules. Western blot analysis provided molecular confirmation of the structural and survival defects observed in *Dhx37*^−/−^ testes. Junctional proteins α-catenin, β-catenin, and N-cadherin were markedly down-regulated relative to WT (Fig. 8E), indicating destabilization of Sertoli-cell adhesion complexes. In parallel, components of the p53 apoptotic cascade were robustly activated: p53 and its downstream effector p21 were up-regulated, while the pro-apoptotic markers BAX and cleaved caspase-3 accumulated (Fig. 8F). Together, these data demonstrate that loss of DHX37 compromises Sertoli-cell junction integrity and triggers a p53-dependent apoptotic program, thereby exacerbating testicular degeneration and infertility.

### *Dhx37* deficiency leads to nucleolar stress

**Fig. 9:**
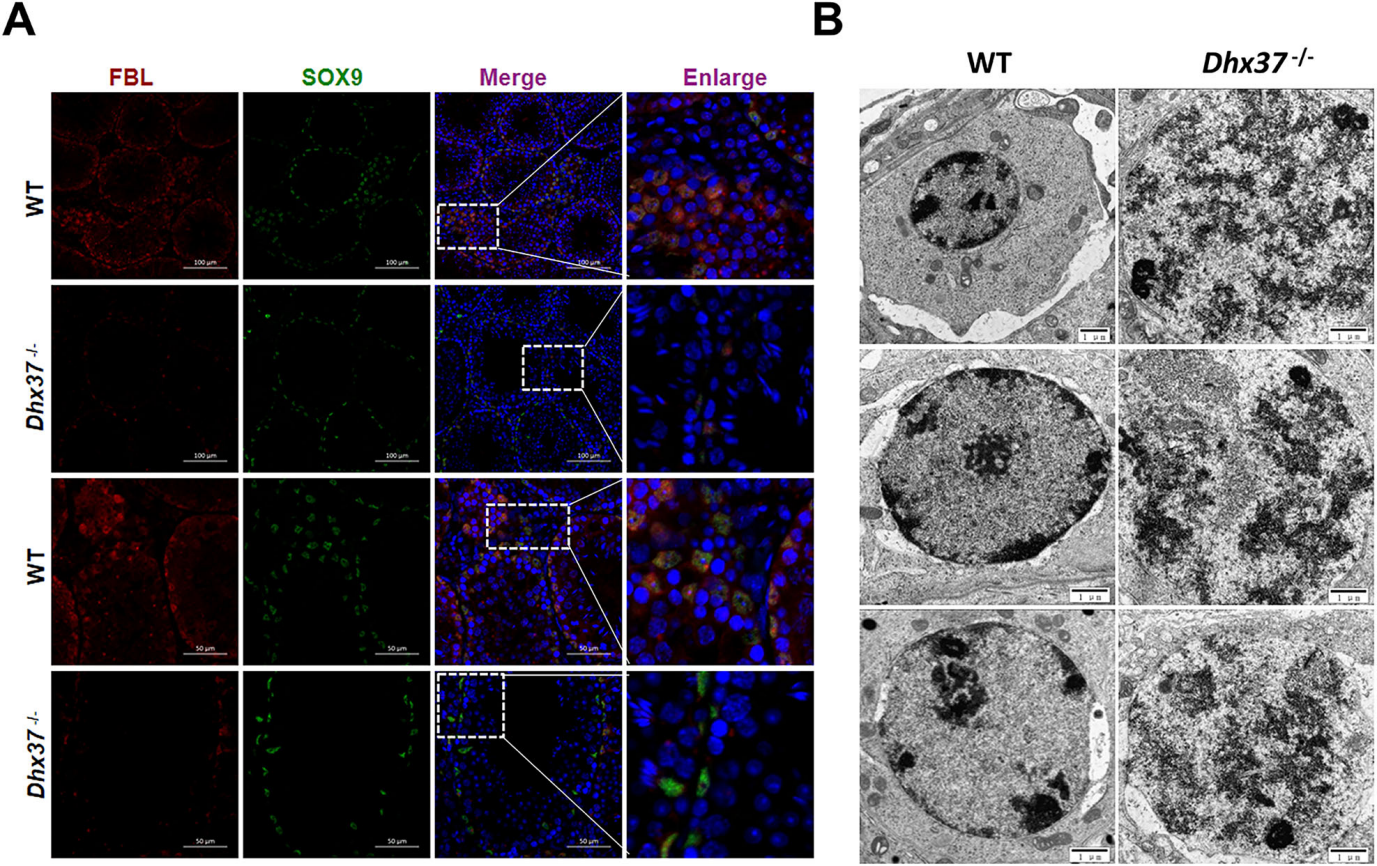
*Dhx37* loss elicits nucleolar stress in Sertoli cells. **A** Immunofluorescence staining of adult (P60) testis sections for the nucleolar marker fibrillarin (FBL, red) and the Sertoli-cell nuclear marker SOX9 (green). Nuclei were counter-stained with DAPI (blue). Right-hand columns show boxed areas at higher magnification. Scale bars, 50 µm. **B** TEM images of Sertoli-cell nuclei. WT nucleoli exhibit the typical tripartite architecture with electron-dense fibrillar surrounded by granular components, whereas *Dhx37*^−/−^ nucleoli appear fragmented, ruptured and markedly reduced in size. Scale bars, 1 µm.

Immunostaining for the nucleolar protein fibrillarin (Fbl), a marker of intact nucleoli, revealed a marked decrease in *Dhx37*^−/−^ testis tissues, indicating nucleolar disruption (Fig. 9A). Loss of nucleolar integrity triggered nucleolar stress, activating pro-apoptotic signaling. To confirm the effect of *Dhx37* knockout on nucleolus structure, TEM was conducted on testes from *Dhx37*^−/−^ and WT mice. *Dhx37* deficiency caused nucleolar fragmentation, rupture, and marked size reduction in Sertoli cells (Fig. 9B). Taken together, our data indicate that loss of *Dhx37* elicits nucleolar stress, which in turn promotes apoptosis and reduces the Sertoli-cell population.

## Discussion

*DHX37* is a kind of RNA helicase that participates in RNA metabolism. It binds to U3 small nucleolar ribosomal RNA (snoRNA) and is essential for ribosome assembly, contributing critically to the maturation of the eukaryotic small ribosomal subunit [16]. Previous studies indicate that *DHX37* is involved in nervous system development and promotes hepatocellular carcinoma cells [23, 24]. Moreover, *DHX37* modulates NF-κB signaling during the anti-tumor immune response [25]. In 2019, a genetic study screened 145 patients with 46, XY DSD, including those with 46, XY gonadal hypoplasia, 46, XY testicular regression syndrome, and severe hypospadias. Pathogenic missense variants in *DHX37* were identified in 13 patients, and statistical analysis demonstrated their significant enrichment in the 46, XY DSD cohort. This study established *DHX37* as the causative gene for 46, XY DSD for the first time [26]. To date, no animal models with gonad-specific *DHX37* deficiency have been reported, and data on *DHX37* expression in human or murine gonads remain limited.

This study combines integrative analyses of public single-cell datasets with RIP-seq and loss-of-function experiments to expand our understanding of *Dhx37* in testicular biology. Three principal insights emerge. *Dhx37* is a ubiquitously expressed helicase in the testis. Single-cell transcriptomes from P3.5 mouse testes reveal that *Dhx37* is detectable in virtually somatic and germ-cell clusters, yet never reaches the transcript intensities of canonical lineage markers, such as Amh or Star. Its expression trajectory mirrors that of the germ-cell factor Dazl rather than the meiosis-restricted Sycp3, indicating that *Dhx37* supports early, lineage-independent program rather than stage-restricted events, such as meiosis.

Additionally, *Dhx37* safeguards ribosome biogenesis and pro-survival signaling in Sertoli cells. RIP-seq of TM4 Sertoli cells shows that *Dhx37* binds mRNAs enriched for focal adhesion, tight junction, and PI3K–Akt pathways, all of which are indispensable for Sertoli–Sertoli and Sertoli–germ cell communication. When *Dhx37* is acutely depleted, we observe a broad collapse of nucleolar and ribosomal gene sets, the selective downregulation of three p53-sensitizing ribosomal protein genes, Rps14, Rps27, and Rpl22 (Supplementary Fig. 1C). Such assembly defects are expected to liberate “free” RPL11 and RPL23 at the protein level, thereby engaging the canonical RPL11–MDM2–p53 nucleolar-stress axis and driving a pro-apoptotic transcriptional response. Concomitantly, key PI3K–Akt components (Pik3r2, Mdm2, Bcl2) are suppressed, amplifying p53 activity and apoptosis. These findings support the emerging view that *DHX37*-linked DSD is a “ribosomopathy” in which ribosome stress drives p53-mediated cell death in pre-Sertoli cells [12, 27, 28]. *Dhx37* loss reprograms alternative splicing, broadening its mechanistic footprint. Nearly one thousand significant AS events—dominated by skipped exons and retained introns—emerge after *Dhx37* knockdown, many of which are located in genes governing cell-cycle checkpoints and DNA damage responses. Because DEAH-box helicases remodel pre-mRNA–spliceosome complexes, these data reinforce *DHX37*’s dual role in ribosome assembly and spliceosome dynamics.

The SRY gene on the Y chromosome initiates the transcriptional program that converts bipotential somatic precursors into Sertoli cells, thereby driving the formation of fully differentiated testicular tissue [29]. Prior to puberty, Sertoli cells predominate in testicular tissue. With pubertal onset, testicular growth accelerates, spermatogonia enlarge, and sperm output rises sharply [30]. The total number of Sertoli cells determines the ultimate capacity for sperm production. Although testicular organogenesis proceeds in the absence of germ cells, spermatogenic cell development is entirely dependent on supporting somatic cells [31]. Overall, the reduced testis-to-body mass ratio, lower circulating testosterone, and diminished sperm counts observed in *Dhx37*^−/−^ mice point to intrinsic Sertoli-cell dysfunction as the underlying cause.

At the organ level, *Dhx37*^−/−^ mice displayed lower testis-to-body mass ratios and reduced sperm counts. At the cellular level, Sertoli and germ cell numbers were markedly diminished. To reconstruct intrinsic developmental trajectories of germ-cell sub-populations, we applied scVelo to the snRNA-seq analysis pipeline [32]. Velocity-based Markov-chain simulations showed that WT germ cells possess highly coherent RNA-velocity vectors converging on a single differentiation endpoint. However, the conditional knockout mice’s germ cells diverged toward novel endpoints, indicating a disruption of the transcriptional program by *Dhx37* loss. RNA velocity analysis revealed a profound re-orientation of gene expression vectors upon *Dhx37* deletion, underscoring *Dhx37* as a pivotal stabilizer of spermatogenesis. During spermatogenesis, *Dhx37* ablation thus compromises transcriptional fidelity in spermatocytes, potentially leading to reduced sperm quality and function*. Dhx37* deletion caused strong fluctuations in gene expression in spermatocytes, particularly between round and elongated spermatocytes. This fluctuation indicates a disruption of gene expression homeostasis during spermatogenesis, which in turn affects the efficiency and stability of spermatogenesis. Consequently, heightened transcriptional noise may impair accurate genetic information transfer, ultimately compromising sperm functionality.

Immunofluorescence assays markedly increased apoptosis and diminished proliferation among testicular germ cells in *Dhx37*^*−/−*^. qRT-PCR analysis corroborated these findings by demonstrating the upregulation of canonical apoptotic mediators, and Western blotting further verified activation of the p53 signaling cascade. We hypothesized that heightened germ-cell apoptosis, together with Sertoli-cell attrition, disrupts the testicular extracellular matrix and compromises the blood–testis barrier (BTB), ultimately depleting the germ-cell pool [33]. Trajectory analysis delineated a single bifurcating lineage for SC maturation (Fig. 8A–C). Mature SCs were characterized by high levels of *Trp73* and *Actn4*, together with housekeeping ribosomal transcripts (*Rpl19*, *Rps3a1*) and DNA-damage regulators (*Cdk7*, *Ppm1d*). Genes central to BTB architecture—*Tjp1*, *RhoA*, *Actg1*—and mitochondrial apoptosis mediator *Cycs* were also up-regulated, underscoring intensified junctional remodeling and stress signaling in *Dhx37*-deficient cells.

Cells at the early pseudotime exhibited enrichment of *Map2k7*, *Ctnnb1*, *Pdgfd*, *Vav3*, *Cntnap2*, *Sesn3*, *Actb*, and *Cftr*, genes that coordinate proliferation, cytoskeletal assembly, and adhesion. As SC number and junction integrity dictate the spermatogenic niche, the concomitant loss of SCs, mislocalization of α-/β-catenin and N-cadherin, and activation of a p53–BAX–cleaved-caspase-3 axis in *Dhx37*^−/−^ testes (Fig. 8D–F) provide mechanistic support for the observed testicular atrophy and infertility [34]. *Dhx37* defects result in BTB dysfunction, erosion of tight and adherens junctions, and progressive seminiferous-tubule atrophy, culminating in reduced testicular mass. BTB disruption affects multiple steps of spermatogenesis, resulting in restricted development and differentiation of spermatogonia, spermatocytes, and spermatids, as well as reduced spermatid numbers [35]. Enhanced death of spermatogonia and spermatocytes further depresses total sperm numbers. Moreover, the loss of supportive Sertoli-cell function limits nutrient supply, impairs spermatocyte survival, and exacerbates the decline in sperm production. [36].

DHX37 is essential for rDNA transcription, playing a crucial role in RNA metabolism and maintaining nucleolar stability [37]. Our RIP-seq analysis in Sertoli cells provided the first clue, revealing that DHX37 binds transcripts enriched in pathways critical for cell survival (PI3K-Akt) and death (p53 signaling, apoptosis). The subsequent RNA-seq and alternative splicing analysis upon Dhx37 knockdown unveiled a profound transcriptional crisis. The concerted collapse of nucleolar- and ribosome-related pathways, together with the selective down-regulation of the p53-sensitizing ribosomal-protein genes Rps14, Rps27, and Rpl22 (Supplementary Fig. 1C), is highly indicative of nucleolar stress—a state that is classically accompanied by the release of “free” RPL11 and RPL23, which then engage the RPL11-MDM2-p53 checkpoint.

To determine whether *Dhx37* defects in Sertoli cells perturb the nucleolus, we found that the number of Fbl labeling was significantly reduced in the *Dhx37*^−/−^ groups [28]. Additionally, TEM detected that the nucleolus structure of Sertoli cells appeared markedly. We propose a coherent model that *DHX37* deficiency disrupts ribosome biogenesis, leading to nucleolar stress and the release of free ribosomal protein RPL11. This activates the classic RPL11-MDM2-p53 surveillance axis [38]. Our data support this pathway: MDM2 is downregulated, leading to the stabilization and transcriptional activation of p53, which in turn upregulates its target genes p21 and the pro-apoptotic effector Bax, while downregulating the survival factor Bcl2 [39]. The disruption of the BTB results in structural damage to and atrophy of the seminiferous tubules, severely affecting spermatogenesis [40]. The result is reduced testicular volume and impaired reproductive development.

The crumpled nucleolar structures in Sertoli cells highlight *Dhx37*’s critical role in nucleolar maintenance. Loss of *Dhx37* is a previously unappreciated axis that links ribosome biogenesis in Sertoli cells to blood-testis barrier integrity and, ultimately, germ cell survival. Consequently, fibrillarin staining and TEM demonstrate gross nucleolar fragmentation in *Dhx37*-deficient Sertoli cells, a hallmark of nucleolar stress. Under nucleolar stress, nucleolar reorganization leads to the redistribution of ribosomal proteins, such as RPL5 and RPL11, and RNA species, including 5S rRNA, into the nucleoplasm [41]. Western blots confirm the stabilization of p53, and both modalities document the robust upregulation of p53 effectors (Cdkn1a/p21, Bax, cleaved caspase-3). The ensuing apoptotic wave explains the striking loss of SOX9-positive Sertoli cells and TRA98-positive germ cells in vivo. At the same time, *Dhx37* deficiency quenching of the pro-survival PI3K–Akt pathway (↓PIK3R2, ↓MDM2) creates a molecular climate in which p53-driven death decisively outweighs compensatory cues (graphical abstract). This redistribution disrupts MDM2’s ability to ubiquitinate p53, leading to p53 stabilization and the subsequent induction of cell cycle arrest or apoptosis through the activation of genes, such as *p21*, *PUMA*, *BIM*, and *NOXA* [42]. The activation of the NF-κB pathway is also a crucial component of the nucleolar stress response [43].

In the absence of *Dhx37*, the breakdown of nucleolar structures and reduction in intact nucleoli in Sertoli cells induce nucleolar stress, resulting in *p53* stabilization, up-regulation of *p21*, cell cycle arrest, and cell death. This mechanistic link aligns with similar observations in other genetic conditions [44]. Nucleolar stress in Sertoli cells reverberates beyond apoptosis. Downregulation and mislocalization of α- and β-catenin, as well as N-cadherin, undermine blood-testis barrier architecture [45]. Similarly, in Diamond-Blackfan anemia (DBA), mutations in ribosomal proteins lead to defects in pre-rRNA processing, altered nucleolar morphology, reduced translation efficiency, and enhanced *p53* stabilization, resulting in the upregulation of *p21* expression and increased apoptosis [46]. *Dhx37* plays a critical role in maintaining this integrity in Sertoli cells, and its disruption leads to significant cellular stress and pathology.

## Conclusions

In this study, we generated the first testis-specific *Dhx37* knockout animal model and observed testicular dysplasia. We identified a critical role for *Dhx37* in testis development and maintenance of nucleolus structure. Genetic defects in *Dhx37* lead to impaired early development of Sertoli cells in the testis.

### Statistical analysis

Sample sizes were determined according to commonly accepted standards in the field and were sufficient to ensure reproducibility of the results. All quantitative analyses were performed under blinded conditions. Animals were allocated to groups based on their genotype (WT or *Dhx37*^−/−^). Within each genotype, individuals were randomly selected for subsequent experiments. The precise sample size (n) for each experimental group is provided in the corresponding figure legends, and no samples or animals were excluded. Normality of the data distribution was assessed using the Shapiro–Wilk test, while homogeneity of variance was examined by Levene’s test. Data meeting these assumptions were expressed as mean ± SD and compared between groups using Student’s *t* test; when assumptions were violated, appropriate nonparametric alternatives were applied. All statistical computations were conducted using R software (version 4.3.2). A two-tailed P value of less than 0.05 was considered to indicate statistical significance.

## Materials and methods

### RNA immunoprecipitation and sequencing RIP-seq

TM4 murine cells (ZQ0091) were expanded under standard conditions and collected at a density of ≈2 × 10⁷ cells per reaction. Pellets were lysed on ice in an equal volume of polysome buffer (100 mM KCl, 5 mM MgCl₂, 10 mM HEPES-KOH pH 7.2, 0.5% NP-40, 1 mM DTT, supplemented with RNase and protease inhibitors). After 5 min on ice, lysates were cleared (15,000 g, 4 °C, 15 min); 40 µL of the supernatant was retained as the input control, and the remainder was used for immunoprecipitation. Protein A/G magnetic beads (100 µL) were equilibrated three times in NT2 buffer (50 mM Tris-HCl, pH 7.4, 150 mM NaCl, 1 mM MgCl₂, 0.05% NP-40) and incubated for 2 h at 4 °C with 5 µg IP-grade anti-DHX37 antibody (Supplementary Table 3). Bead–antibody complexes were resuspended in 850 µL NT2-based IP mix containing RNase inhibitor, 400 µM ribonucleoside-vanadyl complex, 10 mM DTT, and 0.5 mM EDTA. Cleared lysates were added and rotated for 4 h at 4 °C, followed by five washes in ice-cold NT2. RNA was extracted from beads and from input, converted into strand-specific libraries, and sequenced on an Illumina platform (paired-end).

Raw FASTQ data were trimmed with fastp v0.20.1 and depleted of rRNA reads using SortMeRNA v2.1. Clean reads were aligned to the mouse reference genome (mm10) with HISAT2 v2.1.0. Peaks were called using MACS2 v2.2.7.1 with the input sample as background, visualized in IGV, and annotated with BEDTools v2.29.2. De novo and known motifs were identified with HOMER v4.11.1. For enrichment analysis, read counts within peaks were obtained with featureCounts and normalized with the TMM method in edgeR; differential enrichment was assessed directly between the single DHX37-IP sample and its matched input (P < 0.05, |fold-change| > 1). Transcript abundance was quantified with StringTie, and differential expression was likewise calculated by comparing IP to input in edgeR under the same statistical thresholds. Significantly enriched genes were analyzed for GO terms and KEGG pathways in R using hypergeometric testing.

### RNA interference (RNAi)-coupled RNA-seq in TM4 cells

TM4 cells were maintained in DMEM/F-12 supplemented with 10% foetal bovine serum and 1% penicillin/streptomycin at 37 °C in 5% CO₂. For knock-down, cells at 60–70% confluence were transfected with 50 nM *Dhx37*-targeting siRNA (SIDhx37 group) or non-targeting siRNA (negative control, NC group) using Lipofectamine RNAi MAX (Thermo Fisher) according to the manufacturer’s protocol. Forty-eight hours post-transfection, ~3 × 10⁶ cells per biological replicate (n = 3 per group) were harvested by scraping on ice and pelleted at 500 × *g* for 5 min.

Total RNA was isolated with TRIzol™ Reagent (Invitrogen) and quantified on a NanoDrop ND-1000. First-strand cDNA was synthesized with SuperScript II Reverse Transcriptase (Invitrogen), followed by second-strand synthesis in the presence of dUTP using E. coli DNA polymerase I, RNase H (both NEB) and dUTP Solution (Thermo Fisher). After end-repair and A-tailing, Illumina single-index adapters (AMPure XP size selection, target insert 300 ± 50 bp) was ligated. Uracil-containing strands were digested with heat-labile UDG (NEB, M0280).

Expression quantification (FPKM) was performed in StringTie. Differentially expressed mRNAs between SIDhx37 and NC groups were identified with edgeR v3.42.0 (P < 0.05; |fold-change| > 1). Genes meeting these criteria were subjected to Gene Ontology and KEGG pathway enrichment using the R package clusterProfiler with a hypergeometric test (Benjamini–Hochberg-adjusted q < 0.05).

#### Alternative splicing analysis

Raw reads were filtered with Fastp v0.23.4 (phred ≥ 30, length ≥ 36 bp), assessed in FastQC, and aligned to the mouse reference genome (GRCm39, GENCODE vM31) using STAR v2.7.11a in two-pass mode; only uniquely mapped, properly paired reads (MAPQ ≥ 20) were retained. Differential splicing between the SIDhx37 and NC groups (n = 3 each) was identified with rMATS-turbo v4.2.2 (read length = 150 bp, fr-first-strand), which tests five event types—skipped exon, mutually exclusive exon, alternative 5′ and 3′ splice sites, and retained intron. Events with a Benjamini–Hochberg false-discovery rate (FDR) < 0.05 were deemed significant and visualized with rmats2sashimi v2.0. Genes harboring at least one significant event were subjected to GO and KEGG enrichment using clusterProfiler v4.12.0 (q < 0.05), and their overlap with differentially expressed genes was assessed by Fisher’s exact test.

### Generation of *Dhx37* conditional knockout mice

*Dhx37* floxed mice (C57BL/6 based) were generated using CRISPR-Cas technology. *Dhx37*^flox/flox^ mice were then crossed with Amh-Cre transgenic mice to yield *Dhx37*^+/−^ mice. Homozygous conditional knockouts *Dhx37*^−/−^ mice were obtained by mating male mice with *Dhx37*^flox/flox^ females.

### Assessment of fertility

To evaluate the reproductive capabilities and fertility of homozygous *Dhx37*^−/−^ mice, we implemented a standardized mating assay under controlled conditions. Two male *Dhx37*^−/−^ mice, each aged 3 months, were individually housed with two wild-type female mice per cage. Cages were inspected daily for vaginal plugs, and females were subsequently monitored to confirm gestation. The quantity of offspring produced by each mating pair was recorded, providing insight into the fertility impacts of the *Dhx37*^−/−^ genotype.

### Epididymal sperm count

Epididymides from adult *Dhx37*^−/−^ (n = 4) and WT (n = 4) mice were minced in 1 ml 0.9% NaCl, homogenized, and stored at 4 °C for 24 h to release sperm. The homogenate was brought to 1.5 mL with 2% eosin, vortex-mixed, and 10 µL were loaded into a Neubauer hemocytometer. Sperm heads were counted in 25 large squares; the count was multiplied by 10^6^ and by the dilution factor (1.5 ml) to give sperm number per epididymis [47].

### Detection of the serum testosterone levels

Blood was collected from the posterior orbital venous plexus of overnight-fasted 8-week-old mice. Serum testosterone concentrations were quantified using a mouse-specific ELISA kit (Elabscience, E-OSEL-M0003). All assays were performed strictly in accordance with the manufacturer’s guidelines. The kit’s lower limit of detection LODfor testosterone was 0.041 ng mL^−^^1^.

### RNA extraction and quantitative RT-PCR (qRT-PCR)

Total RNA was extracted from mouse testes using the RNAprep Pure Tissue Kit (Tiangen, Cat. DP431). Briefly, 1 μg of total RNA was reverse-transcribed using the PrimeScript RT Reagent Kit with gDNA Eraser (Takara, Cat. RR047A). The reaction mixture for qRT-PCR was prepared as follows: 7.2 μL ddH2O, 10 μL SYBR Premix Ex Taq, 0.4 μL forward primer (10 pmol/μL), 0.4 μL reverse primer (10 pmol/μL), and 2.0 μL template (cDNA/RNA), making a total volume of 20.0 μL. The PCR cycling conditions were as follows: Initial Denaturation: 94 °C for 3 min. Denaturation: 94 °C for 15 s, followed by annealing at 60 °C for 30 s. This step was repeated for 40 cycles. Fluorescence signals were collected at the annealing step (60 °C for 30 s). The relative expression levels of the candidate genes were calculated using the 2^−ΔΔCT^ method. All experiments were repeated three times.

### Isolation of mouse primary Sertoli cells

Seminiferous tubules were dissected, decapsulated, and minced, then incubated at 37 °C for 30 min in 2 mg/mL collagenase IV (Sigma, C5138) and 1 mg/mL DNase I (Beyotime, D7073). After centrifugation (100 × *g*, 1 min, 4 °C) and a PBS wash, the tissue was digested for 20–30 min in 2 mg/mL collagenase I (MCE, 9001-12-1), 1 mg/mL DNase I, and 1 mg/mL hyaluronidase (Yeasen, 20426ES60). A second wash preceded a final 20-min digestion with 2 mg/mL collagenase I, 1 mg/mL DNase I, 2 mg/mL hyaluronidase, and 1 mg/mL trypsin (Sangon, A003702-0100), yielding a suspension enriched for Sertoli cells and type A spermatogonia. Cells were washed with DMEM/F-12, plated in DMEM/F-12 containing 10% fetal calf serum, and cultured at 37  °C in 5% CO₂. After 24 h, residual germ cells were removed by a 2-min hypotonic Tris treatment (20 mM, pH 7.4). Total protein was harvested 3 days later for Western blot analysis [48].

### Immunohistochemistry and H&E staining

Paraffin blocks of mouse testes were sectioned at 5 µm, mounted on SuperFrost Plus slides, and dried at 60 °C for 1 h. Slides were de-waxed in xylene (2 × 10 min) and passed through a descending ethanol series to water. Antigens were unmasked by heating the sections for 7 min in citrate buffer (pH 6.0, 95–100 °C). After endogenous peroxidase was quenched with 3% H₂O₂ (20 min), nonspecific binding was blocked in 5% BSA (30 min, room temperature). Primary antibodies (Supplementary Table S3) were applied overnight at 4 °C. The next day, sections received a biotin-conjugated secondary antibody (1:200, 20 min), followed by HRP-streptavidin and DAB development. Nuclei were counter-stained with hematoxylin, and slides were dehydrated, cleared, and coverslipped. Five random microscopic fields per section were imaged, and staining intensity was scored in ImageJ by an investigator unaware of group assignments.

#### H&E staining

Consecutive paraffin sections were de-paraffinised in xylene and re-hydrated through graded ethanols to distilled water. Slides were immersed in hematoxylin until nuclei appeared blue, briefly rinsed in tap water, then counter-stained with eosin. After dehydration in ascending ethanols and xylene clearing, coverslips were mounted with neutral resin. Representative fields were captured using a Leica bright-field microscope for histological evaluation.

### Immunofluorescent staining

Post-euthanasia, the tissues were immediately excised and fixed in 4% paraformaldehyde (PFA) for an overnight duration. Subsequently, these were embedded and sectioned to a thickness of 3 μm. For permeabilization, the sections underwent treatment with 0.3% Triton X-100 in PBS for 20 min, followed by blocking with 5% BSA in PBS at room temperature for 1 h. The sections were then incubated with specifically diluted primary antibodies, followed by appropriate secondary antibodies. Immunoglobulin G (IgG) served as a negative control for the primary antibody. Nuclear staining was achieved using a final concentration of 1 μg/mL DAPI. Fluorescent images of the stained sections were acquired using confocal microscopy. Quantitative analysis, including the enumeration of gonadotrophoblasts and the measurement of the testicular cross-sectional area, was conducted using ImageJ software.

### Single-nucleus processing and cDNA library preparation

Nuclei were released from P60 mouse testicular cells in Nuclei EZ Lysis buffer (Sigma, NUC-101) supplemented with protease inhibitor (Roche 5892791001) and RNase inhibitors (Promega N2615; Thermo Fisher AM2696) by gentle Dounce homogenization, debris removal (Miltenyi Debris Removal Solution) and filtration through a 20 µm strainer, then resuspended in 1 × PBS/0.07% BSA to 700–1200 nuclei µL^−^^1^. Roughly 8000 nuclei were loaded onto a Chromium controller and processed with the Chromium Single Cell 3′ GEM, Library and Gel Bead Kit v3 (10x Genomics, 1000075) for barcoding, cDNA amplification and library construction. Pooled libraries were size-selected and sequenced on an Illumina NovaSeq 6000 (2 × 150 bp, ≥20000 reads per nucleus). FASTQ files (bcl2fastq v2.20) were processed with Cell Ranger v7.1 against the mm39 reference; nuclei showing 500–5000 genes, ≥500 UMIs, and ≤25% mitochondrial reads were retained. Counts were log-normalized in Seurat v4.4, reduced by PCA (top 10 PCs), clustered with Louvain (resolution 0.5), and visualized by t-SNE. Marker and differential genes (Wilcoxon, adj. p < 0.05) were identified, and GO/KEGG enrichment was analyzed with clusterProfiler.

### Electron microscopy

Immediately after the mice were executed, testicular tissue was extracted and collected in phosphate-buffered saline (PBS). The samples were resuspended in 2.5% glutaraldehyde PBS solution and kept at room temperature for 15 min, incubated on ice for a further 4 h. After three washes with phosphate buffer, the tissues were fixed with 1% OsO_4_ for 40 min. The washed tissue was contrasted with 1% uranyl acetate for 1 h at room temperature and then embedded in 2% purified agar. Agar blocks retaining fixed tissue were dehydrated in a series of different concentrations of ethanol and embedded in epoxy resin. Ultrathin sections (~70 nm) were counterstained with uranyl acetate and lead citrate, and examined using a JEM-1400Flash transmission electron microscope.

## Supplementary information

Suppltal_Fig_WB_originals

SUPPLEMENTARY MATERIAL

## Data Availability

All data generated or analyzed during this study are included in this article and its supplementary information. The RNA-seq and RIP-seq datasets have been deposited in the National Genomics Data Center (NGDC) under accessions CRA030791 and CRA030781, respectively. The validation RNA-seq dataset is available from the GEO under accession GSE309389.
