# Supplementary material for: Multi-omics analysis the effects of Dhx37 deficiency on testis development and nucleolar homeostasis
Source: Cell Death Discov. 2026 Jan 14;12:77. doi: 10.1038/s41420-025-02875-1 (PMC12876971; doi:10.1038/s41420-025-02875-1)

Supplemental Material – Original Blots
Relevant areas for cropped blots in the main Data figures are shown with red boxes

Fig. 3E

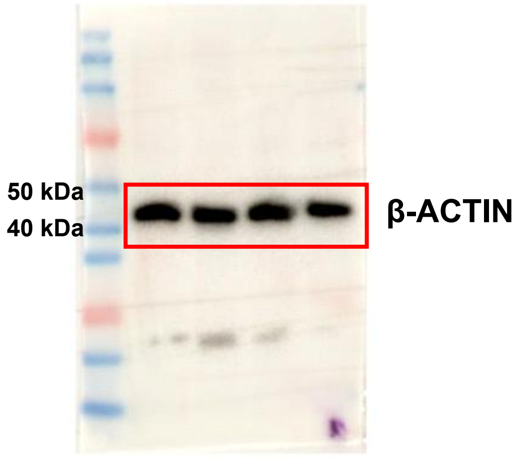

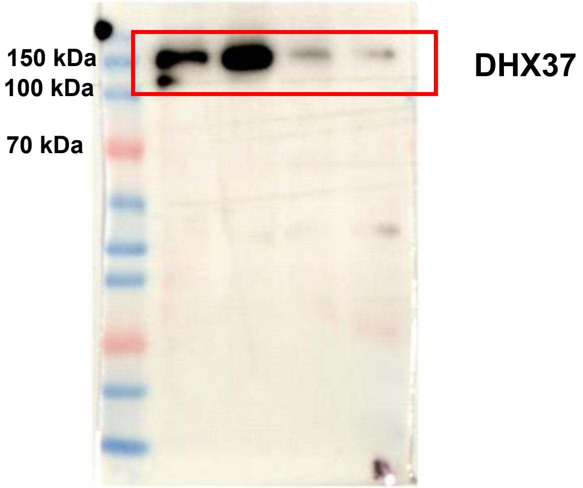

Fig. 5E

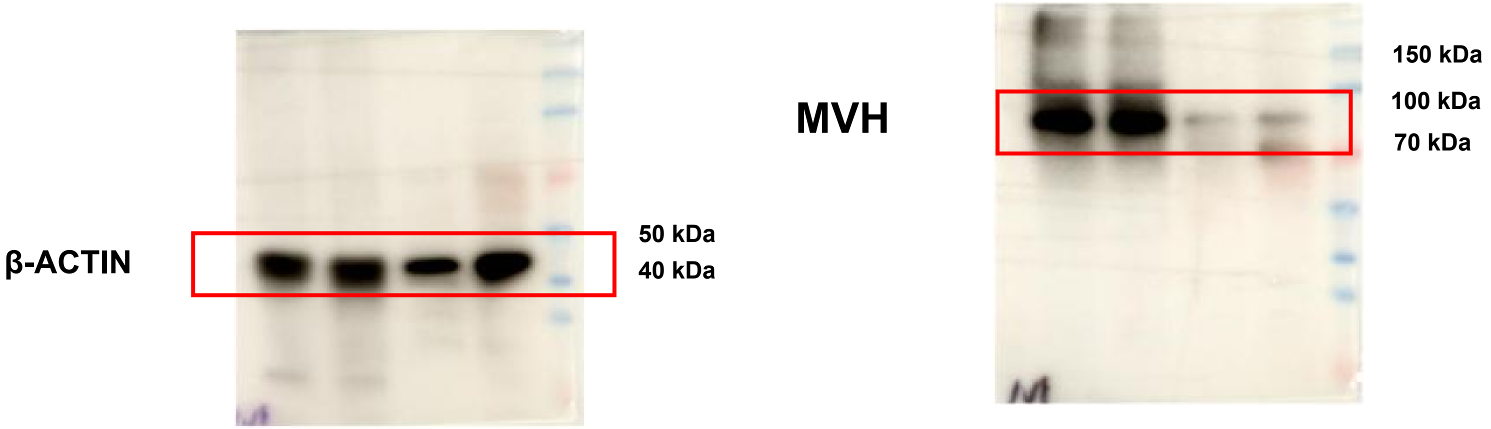

Fig. 5E

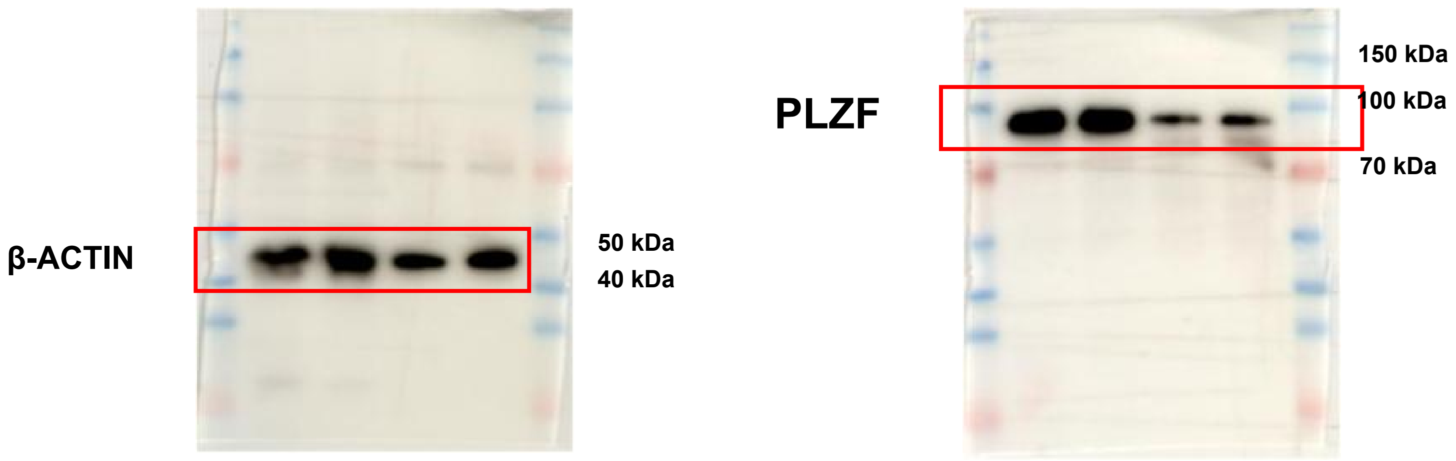

Fig. 5E

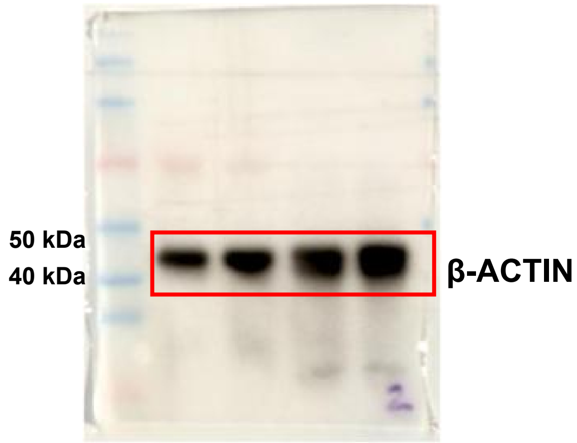

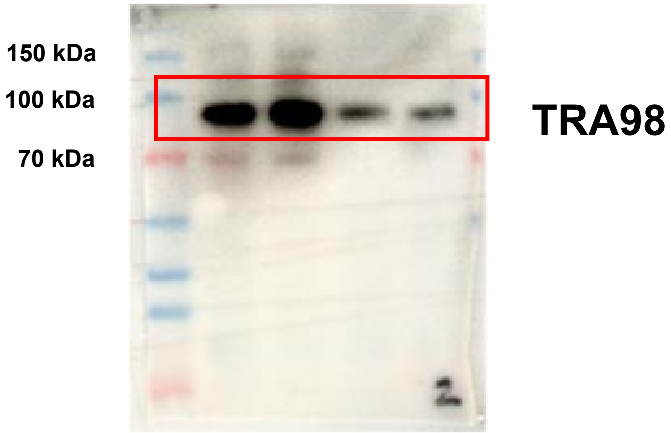

Fig. 7E

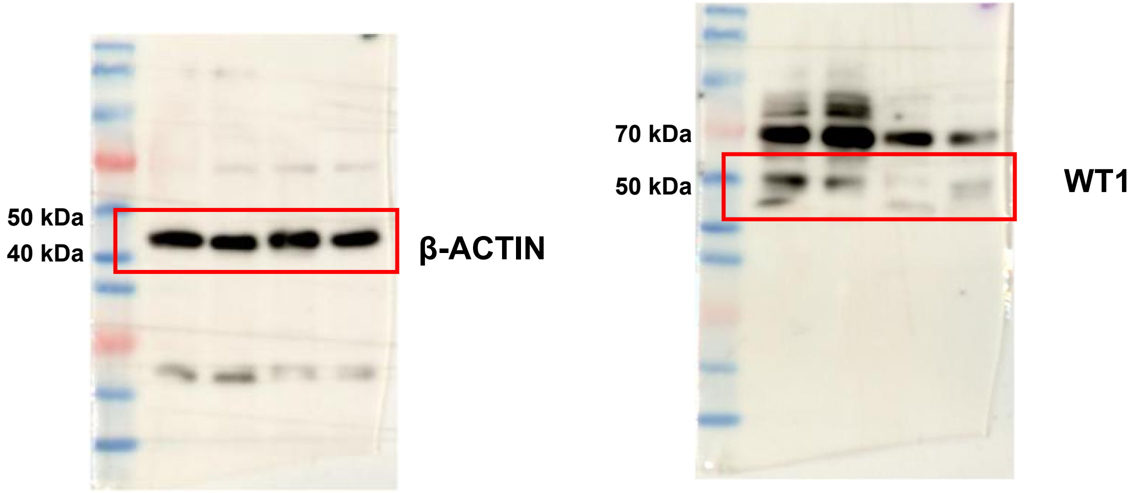

Fig. 7E

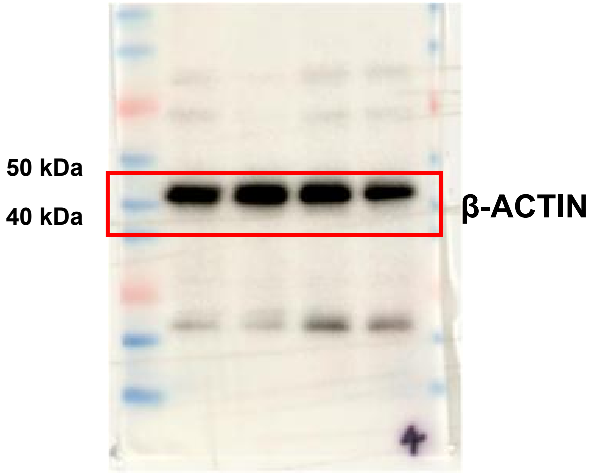

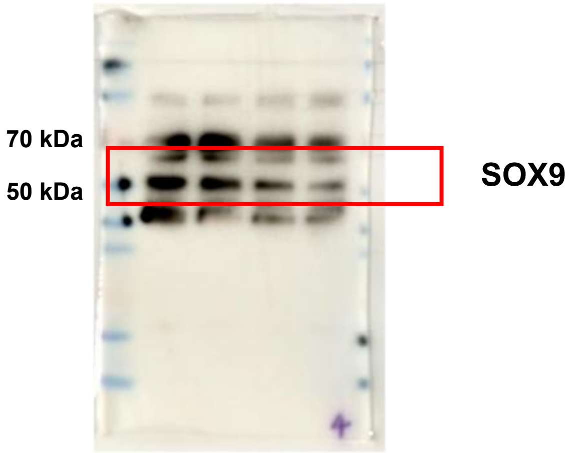

Fig. 8E

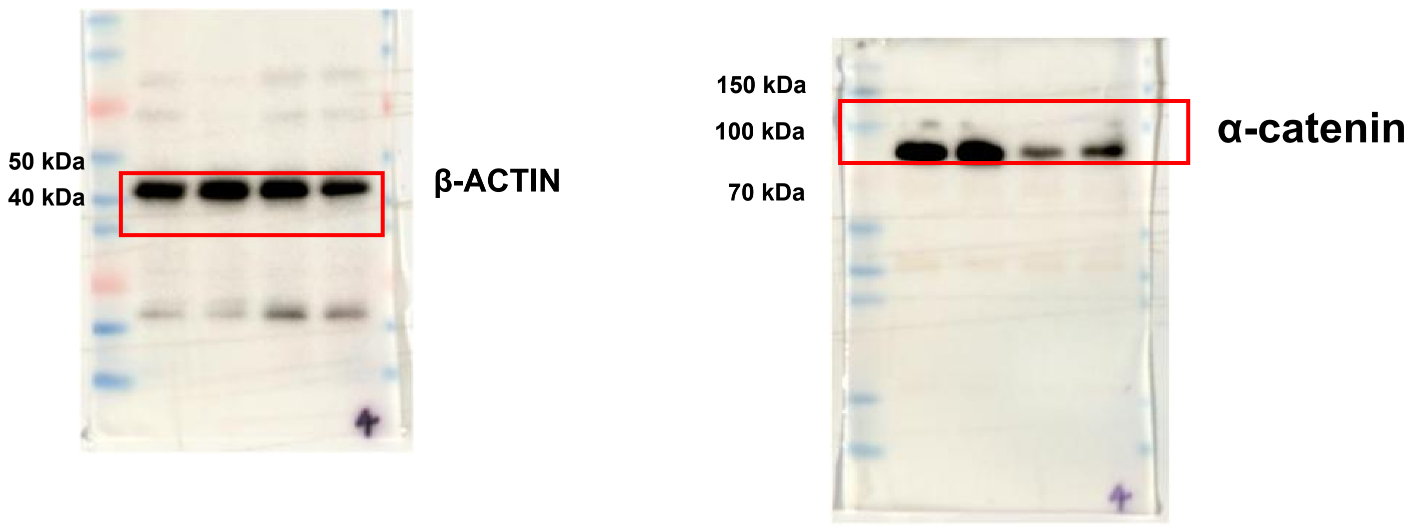

Fig. 8E

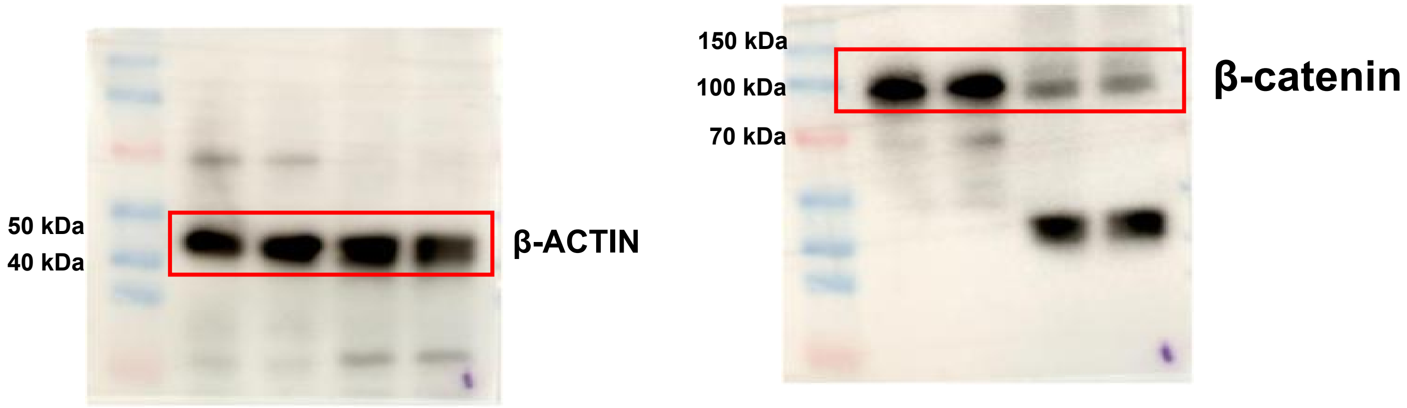

Fig. 8E

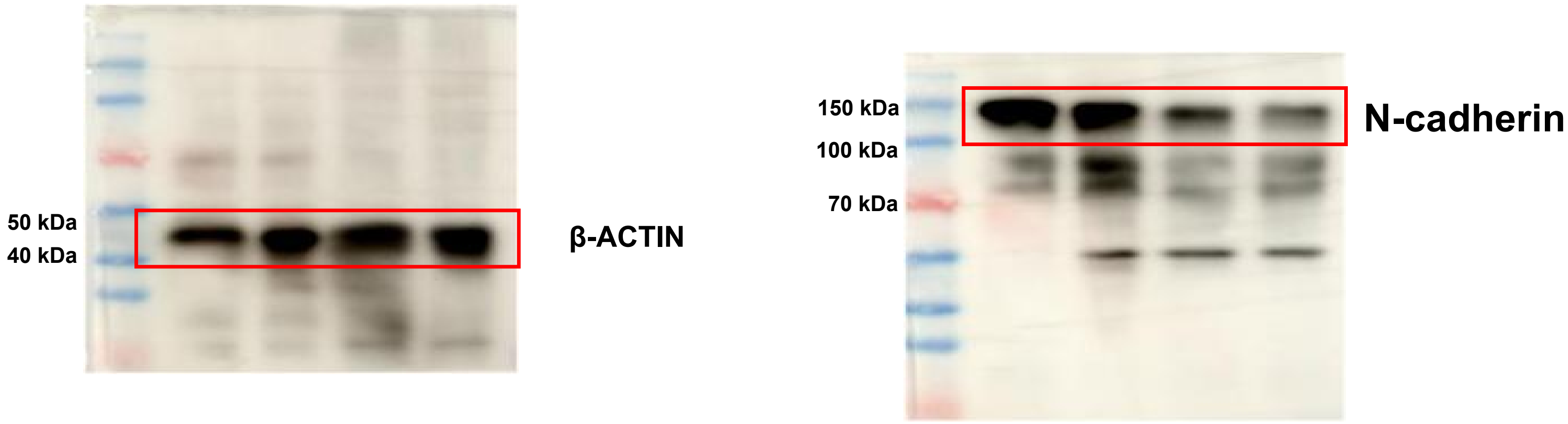

Fig. 8F

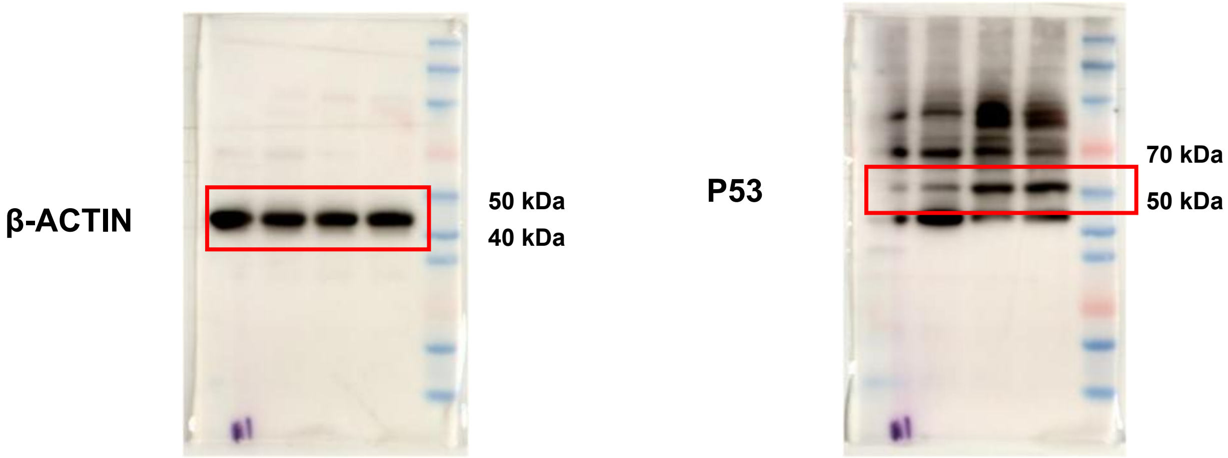

Fig. 8F

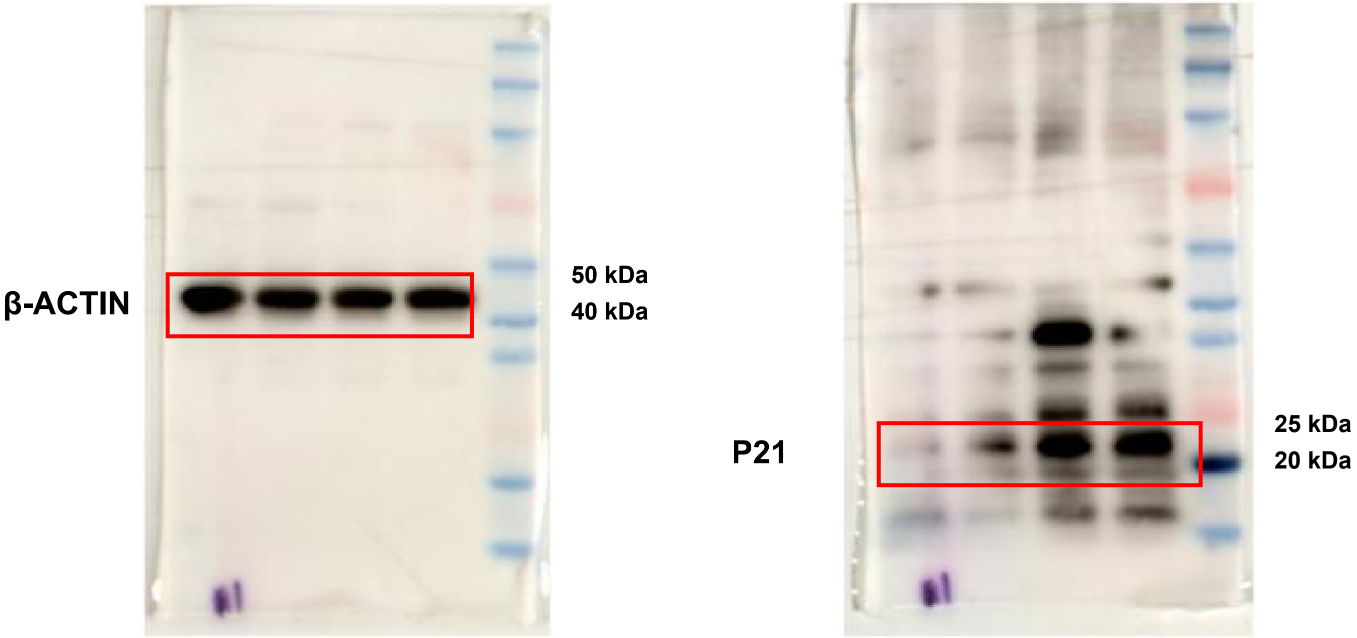

Fig. 8F

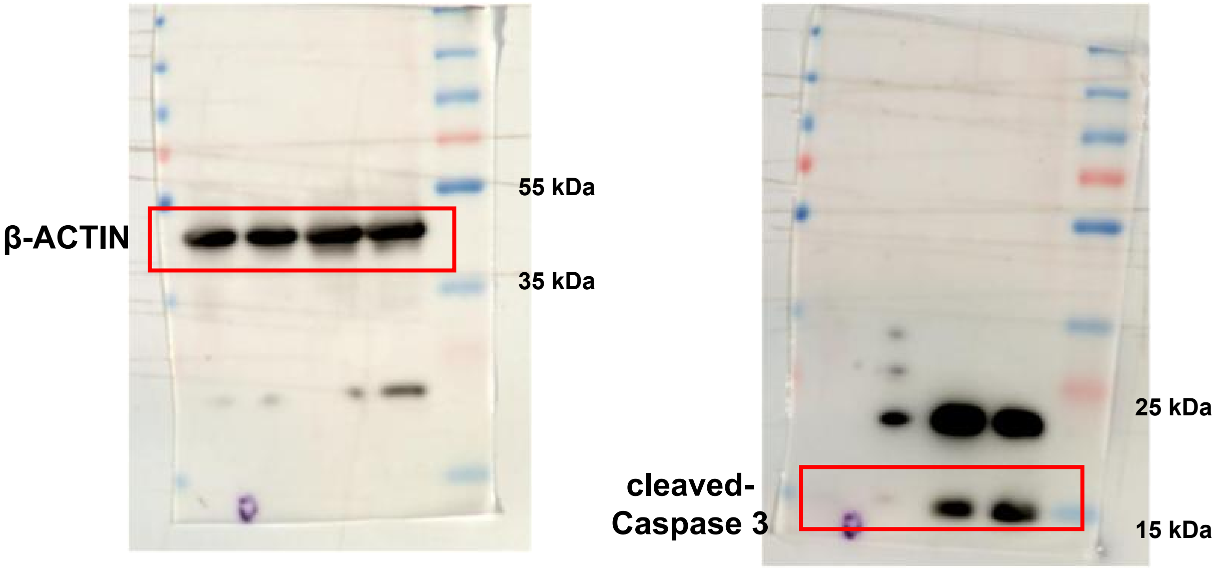

Fig. 8F

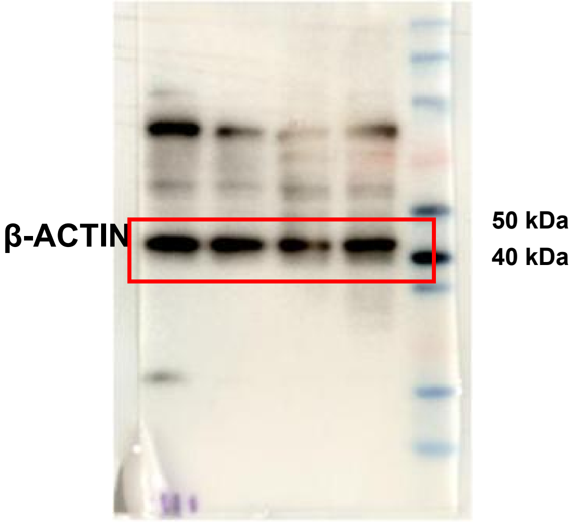

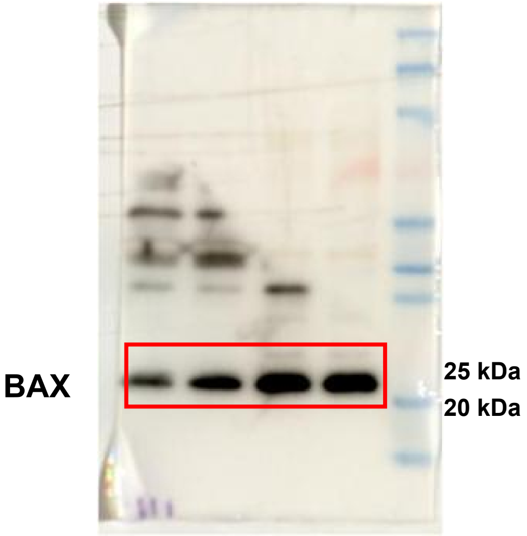

Fig. 8F

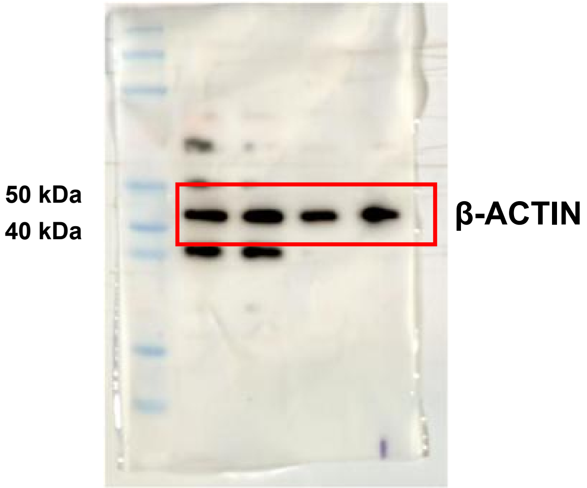

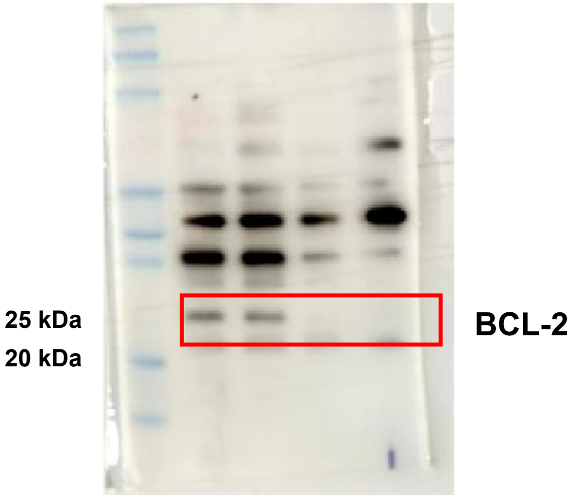

Supplement: Supplementary file 1 — Suppltal_Fig_WB_originals [file 41420_2025_2875_MOESM1_ESM.pdf]
