# Supplementary material for: Multi-omics analysis the effects of Dhx37 deficiency on testis development and nucleolar homeostasis
Source: Cell Death Discov. 2026 Jan 14;12:77. doi: 10.1038/s41420-025-02875-1 (PMC12876971; doi:10.1038/s41420-025-02875-1)
Supplement: Supplementary file 2 — SUPPLEMENTARY MATERIAL [file 41420_2025_2875_MOESM2_ESM.docx]

Supplementary Information

Multi-Omics Analysis the effects of *Dhx37* deficiency on testis development and nucleolar homeostasis

Yuqing Jiang^1^, Jiali Chen^1^, Yanshuang Ren^1^, Wenyuan Peng^1^, Wanjun Shen^2^, Yingyu Zhang^1^, Jie Liu^1^, Liujun Fu^1^, Liping Li^1^, Yujin Ma^1^, Hongwei Jiang^1*^，Huifang Peng^1*^

^1^Henan Key Laboratory of Rare Diseases, Endocrinology and Metabolism Center, The First Affiliated Hospital, and College of Clinical Medicine of Henan University of Science and Technology, Luoyang, China

^2^ National Clinical Research Center for Kidney Diseases, State Key Laboratory of Kidney Diseases, Beijing Key Laboratory of Kidney Disease Research, First Medical Center of Chinese PLA General Hospital, Nephrology Institute of the Chinese People’s Liberation Army, Beijing, China.

* Correspondence: Hongwei Jiang, E-mail: jianghw@haust.edu.cn; Huifang Peng, E-mail: penghuifangsky_@163.com;


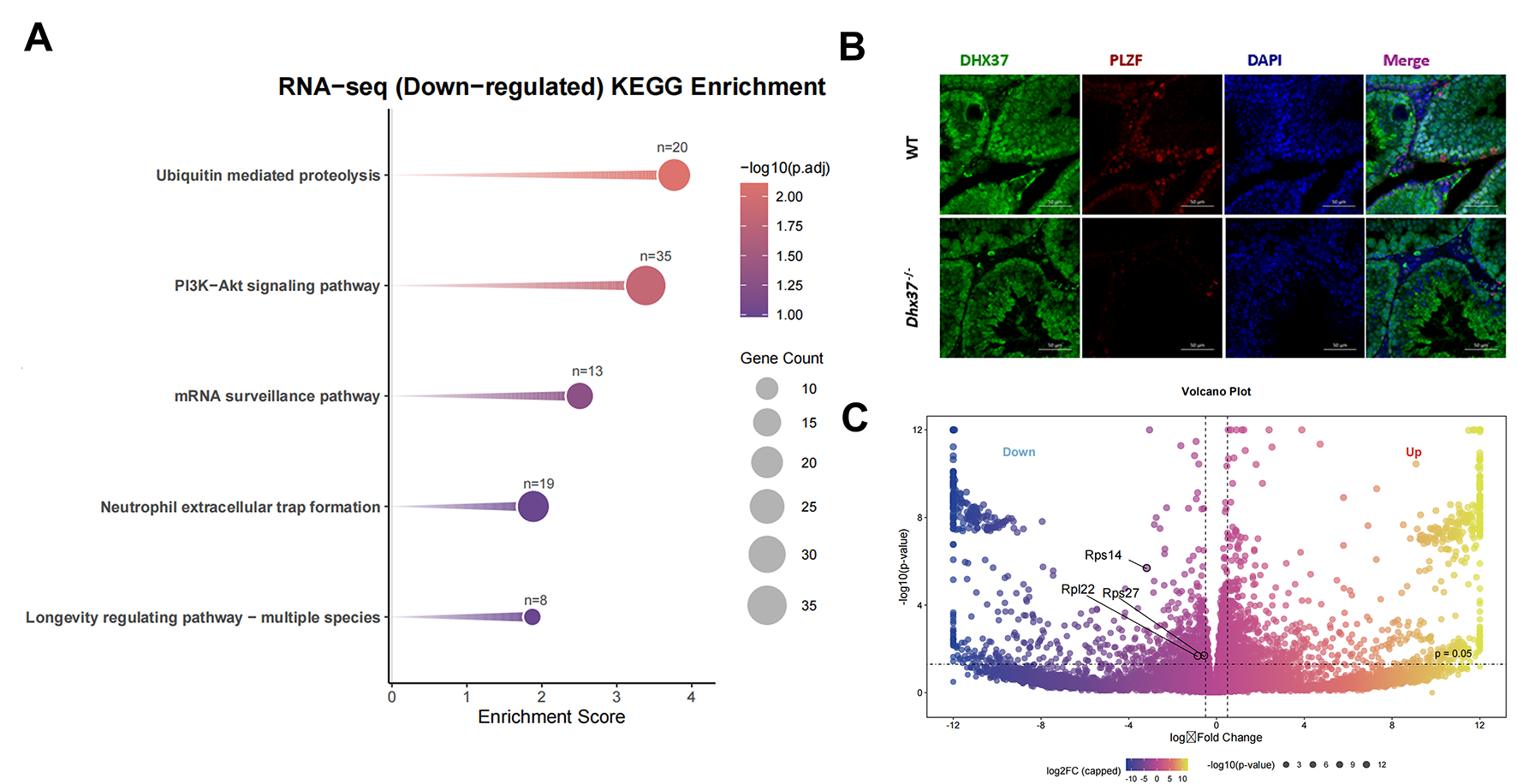


Figure S1. Multi-omic evidence for impaired ribosome-related signalling and germ-cell loss in Dhx37-deficient testes.

(A) KEGG representation analysis of down-regulated transcripts (RNA-seq, *Dhx37*^-/-^ vs. WT). Dot size indicates the number of genes per pathway; colour denotes –log₁₀(adjusted p). The five most significant pathways are shown, headed by “Ubiquitin-mediated proteolysis” and “PI3K–Akt signalling.”

(B) Immunofluorescence of P60 testis sections double-labelled for DHX37 (green) and the undifferentiated spermatogonial marker PLZF (red); nuclei counter-stained with DAPI (blue). *Dhx37*^-/-^ tubules display a near-complete loss of PLZF-positive germ cells. Scale bars, 50 µm.

(C) Volcano plot of the same RNA-seq dataset. The x-axis shows log₂ fold change, the y-axis –log₁₀(p-value). Dashed lines mark |log₂FC| = 0.5 and p = 0.05. Down-regulated ribosomal-protein genes Rps14, Rps27, and Rpl22 are highlighted, supporting a nucleolar-stress signature.

Table S1: Primer sequences used for RT-qPCR

| Gene | Forward 5’-3’ | Reverse 5’-3’ |
| --- | --- | --- |
| *mGapdh* | GTCAAGGCCGAGAATGGGAA | CTCGTGGTTCACACCCATCA |
| *mDhx37* | CGTGTCTTCCTTCCGTGTCA | CTATAGCAGTGGCCTGGCTC |
| *p53* | CCCAGCATCTTATCCGGGTG | GAACCTCAAAGCTGTCCCGT |
| *p21* | TACAAGTATGACCGCGCCTG | CTTCGGTCTCCTGTTCCGAC |

Table S2: siRNA

| siRNA | Forward 5’-3’ | Reverse 5’-3’ |
| --- | --- | --- |
| siNC | UUCUCCGAACGUGUCACG UTT | ACGUGACACGUUCGGAGAATT |
| siDhx37 | CCAGCUCUGUAGUCUUCUCAATT | UUGAGAAGACUACAGAGCUGGTT |

Table S3: Primary and Secondary Antibodies

| Type | Antibody | Application | Dilution | Species | Manufacturer and Catalog Number |
| --- | --- | --- | --- | --- | --- |
| Primary | anti-DHX37 antibody | WB | 1:1000 | Rabbit Polyclonal | Abcam, ab70778 |
|  | p53 Antibody | WB | 1:1000 | Mouse monoclonal | CST, 1C12 |
|  | WT1 Antibody | WB | 1:1000 | Rabbit monoclonal | Proteintech, 82525-1-RR |
|  | SOX9 Antibody | WB | 1:1000 | Rabbit monoclonal | Proteintech, 67439-1-Ig |
|  | TRA98 antibody | WB | 1:1000 | Rat monoclonal | Genetex, GTX00717 |
|  | cleaved-Caspase3 Antibody | WB | 1:1000 | Rabbit Polyclonal | Proteintech, 25546-1-AP |
|  | BCL-2 antibody | WB | 1:1000 | Rabbit monoclonal | Abcam, ab182858 |
|  | BAX antibody | WB | 1:1000 | Rabbit monoclonal | Abcam, ab182733 |
|  | MVH antibody | WB | 1:1000 | Rabbit Polyclonal | Abcam, ab13840 |
|  | PLZF antibody | WB | 1:1000 | Rabbit monoclonal | Proteintech, 66672-1-Ig |
|  | alpha Catenin antibody | WB | 1:1000 | Rabbit polyclonal | Genetex, GTX111168 |
|  | Beta Catenin antibody | WB | 1:1000 | Rabbit polyclonal | Proteintech, 51067-2-AP |
|  | N Cadherin antibody | WB | 1:1000 | Rabbit polyclonal | Abcam, ab18203 |
|  | P21 antibody | WB | 1:1000 | Rabbit polyclonal | Proteintech, 28248-1-AP |
|  | Beta Actin antibody | WB | 1:5000 | Rabbit monoclonal | Proteintech, 81115-1-RR |
|  | SOX9 antibody | IF | 3 µg/ml | Rabbit monoclonal | Abcam, ab185966 |
|  | PLZF antibody | IF | 1:100 | Mouse monoclonal | Santa Cruz, sc-28319 |
|  | MVH antibody | IF | 1 µg/ml | Rabbit Polyclonal | Abcam, ab13840 |
|  | Oct3/4 Antibody | IF | 1:100 | Mouse monoclonal | Santa Cruz, sc-5279 |
|  | TRA98 antibody | IF | 1:400 | Rat monoclonal | Genetex, GTX00717 |
|  | alpha Catenin antibody | IF | 1:1000 | Rabbit polyclonal | Genetex, GTX111168 |
|  | Beta Catenin antibody | IF | 1:200 | Rabbit polyclonal | Proteintech, 51067-2-AP |
|  | N Cadherin antibody | IF | 5µg/ml | Rabbit polyclonal | Abcam, ab18203 |
|  | Ki67 antibody | IF | 1:200 | Rabbit polyclonal | Proteintech, 12225-1-AP |
|  | CYP17A1 antibody | IHC | 1:200 | Rabbit polyclonal | Abcam, ab134910 |
|  | STAR antibody | IHC | 1:200 | Rabbit polyclonal | Proteintech, 12225-1-AP |
| Secondary | HRP-conjugated Goat Anti-Mouse IgG(H+L) | WB | 1:10000 | Goat Anti-Mouse IgG | Proteintech, SA00001-1 |
|  | HRP-conjugated Goat Anti-Rabbit IgG(H+L) | WB | 1:10000 | Goat Anti-Rabbit IgG | Proteintech, SA00001-2 |
|  | CoraLite®488-Conjugated AffiniPure Goat Anti-Rabbit IgG(H+L). | IF | 1:200 | Goat Anti-Rabbit  IgG | Proteintech, RGAR002 |
|  | Cy3–conjugated Goat Anti-Rabbit IgG(H+L) | IF | 1:100 | Goat Anti-Rabbit  IgG | Proteintech, SA00009-2 |
|  | Goat Anti-Rabbit IgG, HRP Conjugated | IHC | 1:200 | Goat Anti-Rabbit IgG | RCA054 |
